# Exposure to Bile Leads to the Emergence of Adaptive Signaling Variants in the Opportunistic Pathogen *Pseudomonas aeruginosa*

**DOI:** 10.3389/fmicb.2019.02013

**Published:** 2019-08-29

**Authors:** Stephanie Flynn, F. Jerry Reen, Fergal O’Gara

**Affiliations:** ^1^BIOMERIT Research Centre, School of Microbiology, University College Cork – National University of Ireland, Cork, Ireland; ^2^School of Microbiology, University College Cork – National University of Ireland, Cork, Ireland; ^3^Telethon Kids Institute, Perth, WA, Australia; ^4^School of Biomedical Sciences, Curtin Health Innovation Research Institute, Curtin University, Perth, WA, Australia

**Keywords:** *Pseudomonas aeruginosa*, evolution, adaptation, pigmented, chronic, quorum sensing, bile, artificial sputum media

## Abstract

The chronic colonization of the respiratory tract by the opportunistic pathogen *Pseudomonas aeruginosa* is the primary cause of morbidity and mortality in cystic fibrosis (CF) patients. *P. aeruginosa* has been shown to undergo extensive genomic adaptation facilitating its persistence within the CF lung allowing it to evade the host immune response and outcompete co-colonizing residents of the lung microbiota. However, whilst several studies have described the various mutations that frequently arise in clinical isolates of *P. aeruginosa*, the environmental factors governing the emergence of these genetic variants is less well characterized. Gastro-oesophageal reflux has recently emerged as a major co-morbidity in CF and is often associated with the presence of bile acids in the lungs most likely by (micro) aspiration. In order to investigate whether bile may select for genetic variants, *P. aeruginosa* was experimentally evolved in artificial sputum medium, a synthetic media resembling environmental conditions found within the CF lung. Pigmented derivatives of *P. aeruginosa* emerged exclusively in the presence of bile. Genome sequencing analysis identified single nucleotide polymorphisms (SNPs) in quorum sensing (*lasR*) and both the pyocyanin (*phzS*) and pyomelanin (*hmgA*) biosynthetic pathways. Phenotypic analysis revealed an altered bile response when compared to the ancestral *P. aeruginosa* progenitor strain. While the recovered pigmented derivatives retained the bile mediated suppression of swarming motility and enhanced antibiotic tolerance, the biofilm, and redox responses to bile were abolished in the adapted mutants. Though loss of pseudomonas quinolone signal (PQS) production in the pigmented isolates was not linked to the altered biofilm response, the loss of redox repression could be explained by defective alkyl-quinolone (AQ) production in the presence of bile. Collectively, these findings suggest that the adaptive variants of *P. aeruginosa* that arise following long term bile exposure enables the emergence of ecologically competitive sub-populations. Altered pigmentation and AQ signaling may contribute to an enhancement in fitness facilitating population survival within a bile positive environment.

## Introduction

Chronic respiratory disease poses a major societal challenge, with the rapid onset of the post-antibiotic era representing a serious threat to the clinical management of infections associated with these conditions (Burney et al., 2015; GBD 2015 Chronic Respiratory Disease Collaborators, 2017). As is often the case, the development of chronic microbial infections results in a shift in clinical regimens wherein these infections are no longer treatable using conventional antibiotics. Therefore, understanding the factors that contribute to respiratory pathogen’s ability to shift from acute to chronic infections is of paramount importance in order to identify alternative interventions and reduce the global reliance on antibiotics.

Cystic fibrosis (CF), resulting from a mutation of the Cystic fibrosis transmembrane conductance regulator (CFTR) gene, is the most common inherited disease in Caucasians affecting over 70,000 people worldwide (Cutting, 2015). The prognosis for this life-limiting autosomal recessive disorder has improved considerably in recent years, largely due to advances in clinical management and the development of new therapeutic interventions. However, challenges remain for the successful implementation of these innovative therapies. Principal among these is maintaining health and lung function in pediatric patients. Lower respiratory infections, even from as early as the first weeks of life, are strongly associated with the development of pulmonary inflammation and bronchiectasis (Stick et al., 2009; Ramsey et al., 2014). Early acquisition of respiratory pathogens including *Staphylococcus aureus* and the subsequent colonization by *Pseudomonas aeruginosa* is associated with clinical decline and increased mortality (Pittman et al., 2011; Caudri et al., 2018). *P. aeruginosa* acquisition often occurs early in life with the median age of initial detection around 2 years of age. Reports of *P. aeruginosa* positive cultures in patients as young as 3 months of age have also emerged (Douglas et al., 2009; Mott et al., 2012). Early eradication has been associated with reduced prevalence of chronic *P. aeruginosa* infections in later life (Taccetti et al., 2005). Therefore, ensuring the successful eradication of *P. aeruginosa* is of paramount importance in CF care.

The pathogen-centric focus to respiratory infections has been challenged somewhat in recent years with the advent of next generation sequencing technologies. Genomics based studies have uncovered richly diverse microbial communities in the lungs of both healthy individuals and hospitalized patients, with perturbations in these populations implicated in many disease conditions (Douglas et al., 2009; Muhlebach et al., 2018). Micro-evolution of pathogens within these diverse communities has also received considerable attention with a growing appreciation for the extent of phenotypic heterogeneity present within these populations (Workentine et al., 2013; Clark et al., 2015; Darch et al., 2015; Grote et al., 2015; Jorth et al., 2015; Williams et al., 2015; Davies et al., 2017). The ability of pathogens to adapt and adopt multiple forms facilitates persistence and colonization in challenging niches such as the lungs of patients with respiratory disease. Mutations in key signaling pathways e.g., LasR are particularly prevalent among *P. aeruginosa* clinical isolates (Hoffman et al., 2009), with pigmented variants also reported in several studies (Mowat et al., 2011; Clark et al., 2015). Furthermore, re-wiring of regulatory networks in these clinical isolates has also been uncovered, suggesting a complex adaptive response to environmental challenges (Feltner et al., 2016; Wang et al., 2018). These changes can lead to phase dependent alterations in virulence (Cabeen, 2014). To enhance early treatment strategies of lung disease in CF, improvements in our understanding of factors that contribute to both pathogen acquisition and dysbiosis of the lower respiratory microbiota is essential.

Bile acids, one of the major components of bile, have been detected in the lungs of patients with respiratory disease, and have been shown to correlate directly with increased morbidity and colonization by *P. aeruginosa* (Zecca et al., 2008; Blondeau et al., 2010; Pauwels et al., 2012; Reen et al., 2014, 2016). This is further supported by several independent studies that show a correlation between bile reflux and pathogen colonization in patients with CF (van der Doef et al., 2009; Palm et al., 2012; Al-Momani et al., 2016), with evidence of increased inflammation and lung impairment also reported (Pauwels et al., 2012). Furthermore, both bile and bile acids have been shown to modulate the behavioral lifestyle of *P. aeruginosa* causing it to adopt a chronic biofilm lifestyle (Reen et al., 2012, 2016). This would appear to occur through transcriptional rewiring of the cell, with changes in virulence systems, signaling molecules, and key metabolic pathways including the glyoxylate shunt observed (Reen et al., 2016). While we have uncovered key insights into the mechanisms through which bile influences the behavior of *P. aeruginosa*, little is known about how the pathogen responds to long term exposure to bile challenge. This would be of particular interest considering the temporal nature of aspiration and its emerging role in shaping the microbiology of the lungs (Dickson et al., 2017).

This study was designed to assess the response of *P. aeruginosa* to long-term bile exposure. Increased production of pyocyanin (PYO) was initially observed and confirmed by transcriptional analysis. Over time, pigmented morphologies were observed exclusively in bile treated cultures. Characterization of these mutants revealed a loss of AQ signaling and PYO production with whole genome sequencing and single nucleotide polymorphism analysis revealing key genetic changes in the pigmented variants. These changes included point mutations in the quorum sensing regulator *lasR*. Although these pigmented mutants retained their response to bile with respect to antibiotic tolerance and swarming motility, they were notably unresponsive for biofilm formation and redox potential response, possibly due to defective AQ production. These findings implicate a role for AQs in the bile induced shift in redox potential with additional unidentified factors likely to be involved in the altered biofilm response to bile on the pigmented mutants.

## Materials and Methods

### Bacterial Culture

All cultures of *P. aeruginosa* were routinely grown in Tryptic soy broth (TSB) media or Luria-Bertani broth (LB) with shaking at 180 rpm at 37°C. PA14 and the transposon mutants described in this study were obtained from the PA14 non-redundant transposon insertion mutant library (Liberati et al., 2006). Strains were maintained on Tryptic Soy agar (TSA) or Luria-Bertani agar (LBA). For the purpose of the antibiotic tolerance assays Mueller-Hinton (MH) agar or broth was used. Bovine bile at a concentration of 0.3% (w/v) was added to media either prior to autoclaving at 105°C for 30 min or after autoclaving by filter sterilization with a 0.2 μm filter.

### Pyocyanin Assay

Overnight cultures of *P. aeruginosa* were adjusted to an OD_600__*nm*_ 0.05 in artificial sputum media (ASM) with and without 3, 0.3, and 0.03% (w/v) bovine bile and incubated at 37°C with shaking at 180 rpm. After 24 and 96 h, test cultures (10 ml) were centrifuged at 5000 rpm for 15 min to obtain a cell free supernatant. Chloroform (3 ml) was added to the cell free supernatant, vortexed and centrifuged for 5 min at 5000 rpm. The bottom blue phase was transferred to a tube containing 2 ml of 0.2 M hydrochloric acid, vortexed, and centrifuged at 5000 rpm for 5 min. The absorbance of the top pink phase was read at OD_520__*nm*_. Calculation of the concentration of pyocyanin (μg/mL) was undertaken according to the following: (OD520 nm × 17.072) × 1.5 to account for the dilution factor following transfer into the acidic phase.

### Pyocyanin and Alkyl Quinolone Extraction

Overnight cultures of *P. aeruginosa* were adjusted to an OD_600__*nm*_ 0.02 in 20 ml TSB with or without 0.3% (w/v) bovine bile and incubated at 37°C with shaking at 180 rpm for 8 h. Culture was centrifuged at 5000 rpm for 15 min to obtain a cell free supernatant with resulting supernatant split into two 10 ml tubes. Pyocyanin was extracted as described above. Alkyl quinolones were extracted by addition of 10 ml of acidified ethyl acetate followed by vortexing and centrifugation for 5 min at 5000 rpm. The top clear phase was transferred to a fresh tube and stored at −20°C overnight. Rotary evaporation was completed to remove the solvent with extracts resuspended in 1 ml of methanol for analysis by thin layer chromatography.

### RNA Isolation and Transcriptional Analysis

Three independent cultures of *P. aeruginosa* strain PA14 were cultured in the presence and absence of 0.3% (w/v) bile (Sigma) in ASM. Untreated and treated samples were inoculated at a starting OD_600__*nm*_ 0.02 and left to grow for 72 h at 37°C. Cultures were centrifuged at 3000 rpm for 10 min with the subsequent pellet resuspended in 1 ml of RNAProtect bacteria reagent (Qiagen). Total RNA was extracted using the RNAeasy kit (Qiagen) according to the manufacturer’s instructions and DNase treated using TURBO DNase (Ambion). cDNA was synthesized using QuantiTect reverse transcription kit (QIAGEN). Real time primers were designed utilizing the Universal Probe Library Assay Design Center (UPL, Roche). RealTime PCR was conducted on a Chromo4 Continuous Fluorescence Detector (MJ Research) using FastStart TaqMAN Probe Master and probes from the Universal ProbeLibrary (UPL, Roche). All gene expression levels are presented relative to the housekeeping gene, *proC*.

### Thin Layer Chromatography

A normal phase silica TLC plate was prepared by immersing in a solution of potassium phosphate monobasic for half an hour. The TLC plate was activated in hybridization oven for 1 h at 100°C. 20 μl of extract were spotted onto the prepared TLC plate with synthetic PQS/HHQ loaded as controls. The plate was developed in 95:5 dichloromethane: methanol and visualized under UV light.

### Cycled Culture of *P. aeruginosa* in Artificial Sputum Media

Artificial sputum media was prepared according to the protocol described by Sriramulu et al. (2005), and consists of mucin, salmon sperm DNA, diethylene triamine pentaacetic acid, sodium chloride, potassium chloride, Tris base, egg yolk emulsion, and casamino acids (Siramulu, 2010). ASM cultures were initiated by inoculation of 2 × 10^6^ cells from an overnight culture of a PA14 *mutS* transposon mutant into 20 ml ASM in the presence and absence of 3% (w/v) bovine bile. As described in Wassermann et al. (2016) the *mutS* transposon mutant was selected due to the mutation in its DNA repair system resulting in enhanced mutation frequency therefore speeding up the rate of mutation and experimental outcome. Cultures were incubated at 37°C static in order to mimic the microanaerobic conditions within CF lung environment for 96 h, with transfer (1:100) into fresh ASM media after mechanical homogenization. Transfers were repeated for 180 days (45 transfers and approximately 315 bacterial generations).

### Colony Morphology Assay

A total of 3 μl of overnight TSB cultures were spotted on to TSA with or without 0.3% (w/v) bovine bile. The spot was allowed to dry at room temperature before incubation at 37°C overnight. Plates were transferred back to room temperature, with colony morphology monitored and recorded over a period of 7 days.

### Pigment Extraction

Strains of interest were inoculated at an OD_600__*nm*_ 0.05 in 50 ml of MH in a 250 ml conical flask for 48 h with visual indication of pigment production. Pigments were successfully extracted by centrifugation of 40 ml of culture at 5000 rpm for 10 min. Ethanol (10 ml) was added to 2 ml of the cell free culture supernatant. The supernatant/ethanol mixture was centrifuged at max speed for a further 10 min. The resulting supernatant was rotary evaporated with compounds collected using methanol. A preparative TLC was undertaken.

### Biofilm Assay

Overnight cultures were adjusted to an OD_600__*nm*_ 0.05 in TSB in the presence and absence of bile. Aliquots of 1 ml were transferred in to 24-well plates and incubated static at 37°C overnight. Biofilm formation was measured by removing spent culture by pipetting. Wells were washed with water by gentle pipetting to remove any unattached biofilm. Attached biofilm was measured by staining for 30 min with 1 ml of 0.1% (w/v) crystal violet. 100% (v/v) ethanol was used to solubilize the crystal violet followed by a measurement of the absorbance at a wavelength of 595 nm.

### Antibiotic Tolerance Assay

Overnight cultures of *P. aeruginosa* were adjusted to 0.5 MacFarland units which is the standard recommendation of the Clinical Laboratory Standard Institute in MH broth. MH agar plates supplemented with or without 0.3% (w/v) bile were uniformly swabbed with culture. E-strips (Thermo Fisher scientific) were placed on to the surface of the agar manually. Plates were left to incubate at 37°C overnight after which the zone of inhibition was recorded.

### Swarming Motility Assay

Swarming motility was measured on 0.6% (w/v) Eiken Agar (Eiken Chemical Tokyo) in the presence and absence of 0.3% (w/v) bile. Sterile toothpicks were used to gently inoculate a single colony onto the surface of the Eiken Agar with minimal pressure. Plates were incubated overnight for 1–2 days with degree of motility visualized and recorded.

### Redox Assay

Overnight cultures of *P. aeruginosa* were adjusted to OD_600__*nm*_ 2.0 with 200 μl of adjusted cultured added to 25 ml of TSB supplemented with 0.01 mg/ml of tetrazolium violet. Cultures were incubated at 37°C shaking at 180 rpm for 24 h. Formazan production was measured by centrifuging 5 ml of culture at 5,000 *g* for 5 min. The supernatant was discarded with the pelleted cells re-suspended in 1.2 ml of dimethyl sulfoxide and centrifuging again at 5,000 *g* for 5 min. The OD_510__*nm*_ was recorded of the cell free supernatant.

### Whole Genome Sequencing

Genomic DNA was extracted from bacterial strains of interest using a Gentra PureGene DNA extraction kit (QIAGEN) with isolated DNA re-suspended in sterile water. Paired end sequencing was conducted by Eurofins Genomics using Illumina MiSeq V3 with 2 × 300 bp reads. Reads were mapped to the *P. aeruginosa* UCBPP-PA14 NC_008463.1 reference genome and delivered as BAM and BAI files. Further sequence analysis for SNP identification was conducted using the Integrative Genome Viewer software platform using the reference strain UCBPP-PA14 and the more recent PA14 Or genome sequence (NZ_LT608330.1). This Whole Genome Shotgun project has been deposited at DDBJ/ENA/GenBank under the accession SZXO00000000 (red pigmented mutant), SZXP00000000 (brown pigmented mutant), and SZXQ00000000 (yellow pigmented mutant).

## Results

### Pyocyanin Production Is Elevated in the Presence of Bile in ASM

Bile has emerged as a key host factor capable of modulating behavioral changes in the life cycle of the opportunistic respiratory pathogen *P. aeruginosa* (Reen et al., 2012, 2016). Transcriptomic analysis conducted in the presence of bile has previously revealed changes in the expression of an array of quorum sensing and PYO related genes (Reen et al., 2016). Furthermore, the observed shift in the redox status to a more reduced level was also notable considering the inherent relationship between PYO (and other phenazine pigments) and the maintenance of redox homeostasis within the cell. Therefore, we investigated the impact of bile on PYO production in *P. aeruginosa* PA14 grown in ASM, a growth medium that is known to mimic conditions found within the CF lung environment.

**FIGURE 1:**
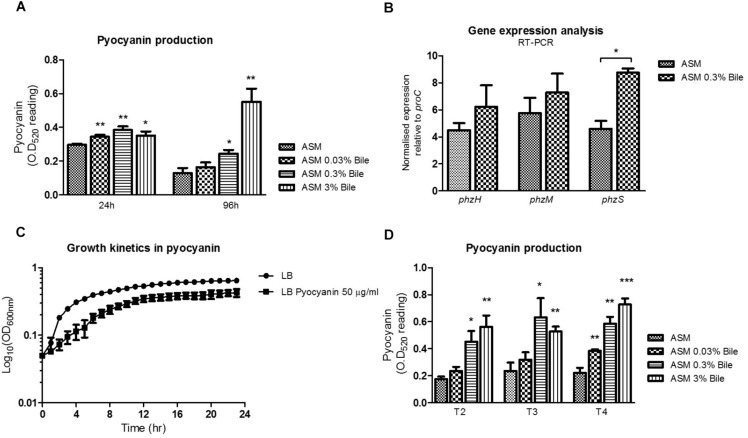
**(A)** The phenazine PYO is elevated in ASM supplemented with a range of bile concentrations. **(B)** Enhanced PYO production is underpinned by increased *phzS* expression, the final step of the PYO biosynthetic pathway, as measured by RT-PCR analysis. No significant change in gene expression was observed for *phzH* or *phzM*, the two other central genes in PYO biosynthesis. **(C)** PYO, at a concentration of 50 μg/mL reduces the growth rate and biomass of *Pseudomonas aeruginosa.* **(D)** Serially cycling *P. aeruginosa* in ASM supplemented with bile confirms the consistent up-regulation of PYO production over time. Data is the mean of at least three independent biological replicates. Statistical analysis was performed by Student’s *t*-test (^∗^*p* ≤ 0.05; ^∗∗^*p* ≤ 0.01; ^∗∗∗^*p* ≤ 0.001).

Bile, at various sub-inhibitory concentrations was shown to significantly increase production of PYO in *P. aeruginosa* in ASM when compared to untreated cultures (Figure 1A). Induction was evident at 24 h incubation but was most pronounced at 96 h in the presence of 3% (w/v) bile. The range of bile concentrations tested are physiologically relevant, with 0.03–0.3% (w/v) bile previously shown to elicit a behavioral switch in *P. aeruginosa* (Reen et al., 2012). Higher concentrations of 3% would be encountered in the gastrointestinal tract where *P. aeruginosa* may possibly be conditioned prior to aspiration into the lungs of at risk patients (Reen et al., 2012). Induction was evident at 24 h incubation but was most pronounced at 96 h in the presence of 3% (w/v) bile. At 24 h, concentrations of pyocyanin went from 7.59 μg/mL in the absence of bile to 8.82, 9.85, and 9.01 μg/mL in the presence of 0.03, 0.3, and 3% (w/v) bile, respectively. At 96 h, concentrations of pyocyanin went from 3.31 μg/mL in the absence of bile to 4.22, 6.27, and 14.13 μg/mL in the presence of 0.03, 0.3, and 3% (w/v) bile, respectively. Gene expression analysis as measured by qRT-PCR on three branch point PYO biosynthetic genes (*phzH*, *phzM*, and *phzS*) revealed a significant increase in the expression of *phzS* in bile treated ASM cultures (Figure 1B). A trend toward increased expression of *phzH* and *phzM* was also observed although it did not reach statistical significance. *phzS*, encoding a flavin-containing monooxygenase, is the final gene of the PYO biosynthetic pathway responsible for the conversion of 5-methylphenazine-1-carboxylic acid betaine to PYO. The increased expression of this gene may underpin the overproduction of PYO following *P. aeruginosa* exposure to bile.

Growth curve analysis was undertaken to investigate the impact that the accumulation of PYO within the microbial community may have on growth rate. An upper range concentration of 50 μg/mL, which may be representative of the accumulation of elevated levels of pyocyanin observed in bile treated cultures, was chosen for these experiments. A recent study by Meirelles et al. has shown that elevated levels of PYO can have both beneficial and detrimental effects on *P. aeruginosa* populations (Meirelles and Newman, 2018). Consistent with these findings we found that a concentration of 50 μg/mL of PYO reduced the growth rate and resulting biomass of *P. aeruginosa* (Figure 1C). To investigate the effects of longer-term bile exposure on PYO production, *P. aeruginosa* was serially transferred into fresh ASM at 96 h intervals. Subsequent PYO analysis following three rounds of 96 h growth cycles confirmed that PYO levels remain elevated following the prolonged exposure to bile (Figure 1D). Therefore, it was essential to understand the mechanism(s) by which *P. aeruginosa* might adapt to these elevated, toxic levels of PYO which would potentially represent a selective pressure for the evolution of *P. aeruginosa* within a bile positive lung environment.

### Hyper-Biofilm Forming Pigmented Variants of *Pseudomonas aeruginosa* Emerge Exclusively in the Presence of Bile

**FIGURE 2:**
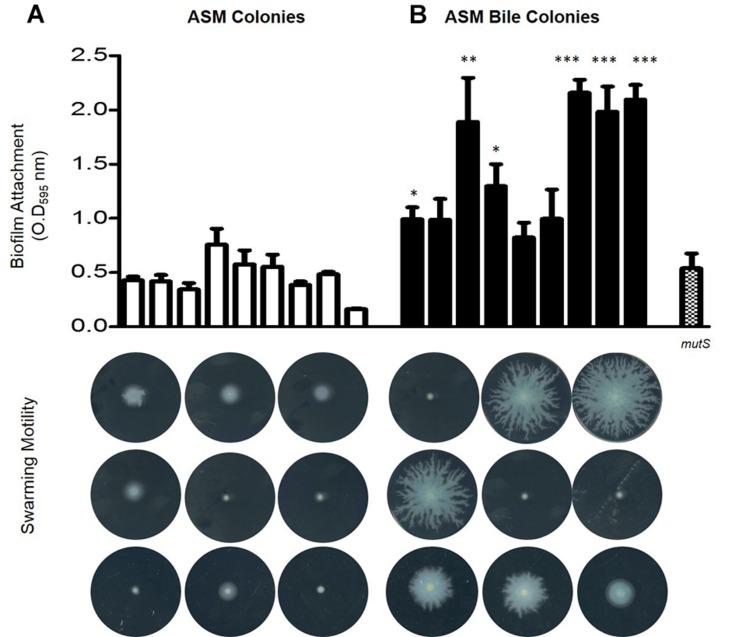
Phenotypic analysis encompassing biofilm formation and swarming motility in 9 colonies from three independent biological replicates isolated from **(A)** untreated ASM and **(B)** bile treated ASM. Data is the mean of at least three independent biological replicates. Statistical analysis was performed by Student’s *t*-test (^∗^*p* ≤ 0.05; ^∗∗^*p* ≤ 0.01; ^∗∗∗^*p* ≤ 0.001).

Upon confirmation that PYO levels remained elevated upon sequential transfer of *P. aeruginosa* in ASM supplemented with bile, serial transfers were performed for a duration of 6 months encompassing approximately 45 transfer events (*n* = 3). Biofilm analysis of 9 randomly selected end-point isolates from untreated ASM and 9 randomly selected end-point isolates from bile treated ASM isolated from 3 independent biological replicates revealed that bile treated ASM isolates displayed elevated biofilm levels relative to untreated ASM isolates (Figure 2). Consistent with the findings of Davies et al. (2017), swarming motility appeared to be dramatically reduced in the untreated ASM colonies. However, there was an apparent selection for the maintenance of swarming motility in several of the bile treated ASM colonies. These findings were further confirmed by larger scale analysis of randomly selected isolates taken from the final replicate of the adaptive experiment encompassing 48 colonies from bile treated ASM and 48 colonies from untreated ASM. The same pattern of enhanced biofilm and retention of swarming motility was observed in colonies selected from the bile treated ASM samples when compared with those selected from ASM alone (Supplementary Figures 1, 2).

Colony morphology analysis revealed the emergence of uniquely pigmented variants of *P. aeruginosa* derived from ASM in the presence of bile, with yellow, red and brown derivatives observed (Supplementary Figures 3A–D). The yellow pigmented variant was the first to emerge, followed by the brown and red pigmented variants, respectively. The yellow pigmented variant became the most dominant member of the community representing approximately 60% of the population with the brown and the red variants comprising approximately 10 and 5% of the population, respectively, upon completion of the experiment. Interestingly, though the brown and red derivatives were maintained in the community, they did not significantly increase in abundance with time. This suggests that there may be some fitness cost preventing them from outcompeting the progenitor wild-type phenazine producing genotype within the population. In contrast to the bile treated ASM cultures, the majority of colonies obtained from untreated ASM appear to either retain green pigmentation, most likely a result of intact phenazine production, or lose pigmentation completely (Supplementary Figure 3A). Furthermore, colony morphologies of the untreated ASM cells appeared larger and more rugose than their bile treated counterparts, which were smaller and more smooth (Supplementary Figure 3A).

### Genotypic Profiling of the Pigmented Variants

**FIGURE 3:**
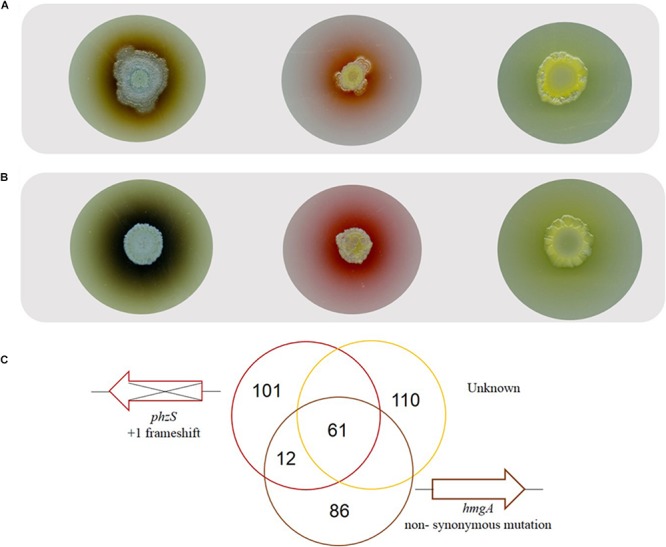
**(A)** Representative colony morphology analysis of the brown, red, and yellow pigmented variants on TSB agar and **(B)** TSB agar supplemented with bile highlighting pigment production with further induction in the presence of bile. **(C)** Whole genome sequence analysis on representative pigmented isolates with sequence comparison analysis undertaken with PA14 or as the reference strain.

As the pigmented derivatives emerged exclusively in the presence of bile, red pigmented variants (*n* = 3), brown pigmented variants (*n* = 3), and yellow pigmented variants (*n* = 3) were selected for further analysis in order to elucidate their functional importance in the presence of bile. Colony morphology analysis confirmed the stable production of the brown/red/yellow pigment which appears to be extruded from the colony (Figure 3A). However, in the presence of bile there appears to be a further increase in the production of these pigments. Therefore, though a mutational event likely underpins the production of these alternative pigments, the intact regulatory pathway governing the regulation of pigment production appears to still be responsive to bile (Figure 3B).

Whole genome sequencing analysis was conducted on a representative red, brown and yellow pigmented isolate. Sequence validation following PCR amplification using gene-specific primers was carried out on selected target genes in the *mutS* ancestral strain. While all three isolates were found to have 281 SNP’s in common when using the UCBPP-PA14 reference strain, this number was reduced down to 61 SNP’s when using PA14 Or as a reference strain (Figure 3C). This finding is important when considering the use of these reference genomes for SNP analysis of *P. aeruginosa* clinical isolates and highlights the potential importance of using multiple reference genomes in clinical genome analysis. The genetic events within each of the individual isolates described below with indels in coding regions representing the most important category as these will definitively alter the protein (Table 1). These indels are further outlined in Supplementary Figure 4.

**TABLE 1:** Genetic events present in all three pigmented variants, the brown pigmented variant alone, the red pigmented variant alone, and the yellow pigmented variant alone.

	**Coding regions**			**Intergenic**	
	**Indels**	**Nucleotide substitutions**		**Indels**	**Nucleotide substitutions**
		**Synonymous**	**Non-synonymous**		
Common	11	8	27	8	23
Brown	14	8	53	5	8
Red	7	23	51	4	16
Yellow	10	20	71	3	6

Within the genome of the brown pigmented variant a single base pair substitution at nucleotide 984 of a T to a C was identified in the *hmgA* gene. This non-synonymous substitution results in a change of the amino acid phenylalanine (TTC) to leucine (CTC). This single base pair mutation was also located in the *hmgA* gene of the two other brown pigmented variants tested, as confirmed by sequencing of PCR amplicons (Supplementary Figure 5). Loss of function mutations in *hmgA* have previously been shown to result in production of the brown polymeric pigment pyomelanin by *P. aeruginosa* (Rodriguez-Rojas et al., 2009). However, further characterization will be required to definitively attribute production of the brown pigment to the SNP identified in this study.

Within the genome of the red pigmented variant sequence analysis revealed the insertion of a C nucleotide at position 125 of the *phzS* encoded protein. The production of a red pigment in *P. aeruginosa* has previously been reported for *phzS* mutants, suggesting that this insertion underpins the pigmentation the red variant (Gibson et al., 2009). The insertion of a C nucleotide at position 125 resulted in a+1 frameshift mutation which was confirmed by PCR sequencing and extended to all other red variants tested (Supplementary Figure 5). Interestingly, subsequent plating of red pigmented variants consistently led to loss of pigmentation in a subset of colonies. Sequencing of these colonies revealed reversion back to the original ancestral sequence. This is consistent with previous studies describing a high frequency of reversion for +1 frameshift mutations in a *mutS* mutant (Smania et al., 2004). This finding validated that insertion of the single C nucleotide into the *phzS* sequence was responsible for production of the red pigment in these mutants. Additionally, pigment extraction and preparative TLC analysis from a *phzS* transposon mutant and the red derivative further reinforced these findings with an RF value of 0.92 obtained for both the *phzS* mutant and the red pigmented isolate. While the potential causative mutations could be identified for the red and brown pigmented variants, no likely mutation could be located that could underpin production of the yellow pigment. The *phzM* gene and upstream regions including the promoter region were unchanged in all strains. It is possible that another mutation within the genome has affected the expression of *phzM* and therefore production of the yellow pigment could still be attributed to altered *phzM* expression. However, previous studies have also shown that insertional mutagenesis of *rhlI* results in yellow pigmentation, possibly as a consequence of increased pyoverdine production (Orlandi et al., 2015). Therefore, elucidating the origin of the yellow pigment identified in this study will require further mechanistic investigation.

### Phenotypic Profiling of Pigmented Mutant Reveals Altered Biofilm Response to Bile

**FIGURE 4:**
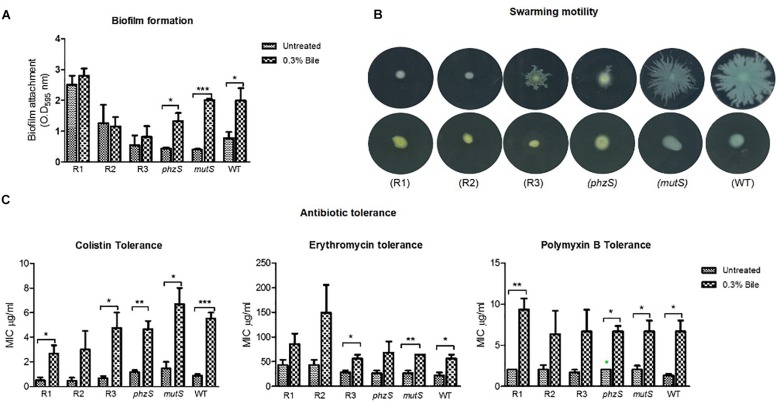
Phenotypic profiling in the presence and absence of bile of the red pigmented derivatives, *phzS* and *mutS* transposon mutants and the PA14 WT strain for **(A)** biofilm formation. **(B)** Swarming motility. **(C)** Antibiotic tolerance. Data is the mean of at least three independent biological replicates. Statistical analysis was performed by Student’s *t*-test (^∗^*p* ≤ 0.05; ^∗∗^*p* ≤ 0.01; ^∗∗∗^*p* ≤ 0.001).

As the strongest evidence of a causative mutation was found in the red pigmented variant, three representative isolates of this variant were phenotypically profiled in tandem with a *phzS* transposon mutant, the ancestral progenitor *mutS* transposon mutant and the PA14 WT strain. Key phenotypes associated with the chronic lifestyle, including biofilm formation, swarming motility, and antibiotic tolerance, were tested (Figure 4). Importantly, all three phenotypes were previously shown to be modulated in response to bile where biofilm and antibiotic tolerance were both increased, while swarming motility was repressed (Reen et al., 2012, 2016). As the red pigmented mutants arose solely in the presence of bile supplemented ASM, we reasoned that these mutants may respond differently to the wild-type strain when challenged with this important host factor.

In respect of biofilm formation, the bile-induced biofilm formation evident in the wild-type and *mutS* transposon mutant was not observed in the red pigmented isolates. One of the three red pigmented mutants tested exhibited a higher level of biofilm formation when compared with the wild-type and *mutS* mutant. This is expected on the basis of the high levels of biofilm formation in the bile treated ASM colonies. However, there was no significant increase in biofilm formation in any of the red variants in the presence of bile (Figure 4A). The *phzS* mutant exhibited a comparable increase in biofilm to the wild-type and *mutS* mutant, suggesting that pigment production alone is not responsible for the loss of enhanced biofilm formation in the presence of bile. All three red pigmented variants were found to be swarming mutants, as was the case for the *phzS* transposon mutant (Figure 4B). In contrast, the *mutS* transposon mutant exhibited wild-type swarming motility which was suppressed in the presence of bile. The increase in tolerance to macrolide and polymyxin antibiotics was also apparent in the red pigmented variants while also being retained in the *phzS* mutant (Figure 4C). However, the trend toward increased antibiotic tolerance did not reach significance in the R2 red pigmented variant. Notwithstanding their increased tolerance to colistin in the presence of bile, it was notable that the red pigmented mutants were themselves more susceptible to colistin when compared directly with the mutS mutant and wild-type strain. The differential biofilm response between the red derivatives and the *phzS* transposon mutant further suggests that other factors may be involved in mediating enhanced biofilm in response to bile in *P. aeruginosa*.

**FIGURE 5:**
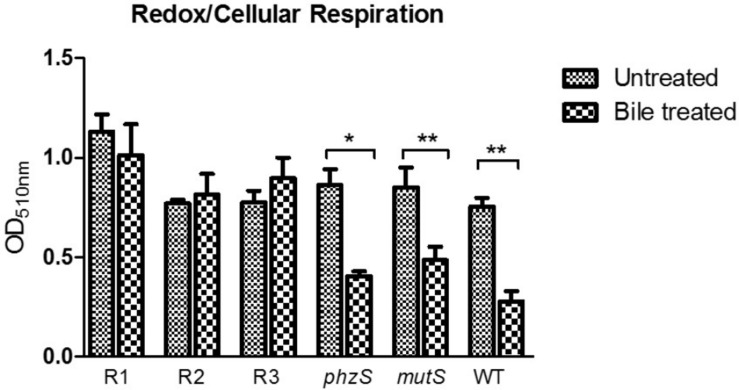
Tetrazolium violet redox assay revealing the lack of repression of redox in the presence of bile in the red pigmented variants with a repression of redox evident in the *phzS* mutant. Data is the mean of at least three independent biological replicates. Statistical analysis was performed by Student’s *t*-test (^∗^*p* ≤ 0.05; ^∗∗^*p* ≤ 0.01).

The perturbations of redox homeostasis appear intricately connected to the ability of *P. aeruginosa* to thrive in bile. Transcriptional profiling has previously shown changes in the expression of genes encoding metabolic pathways, while the redox profile was shown to be repressed in the presence of bile (Reen et al., 2016). Furthermore, the functional role of pigments within *P. aeruginosa* is linked to the maintenance of redox homeostasis within the cell (Dietrich et al., 2006; Price-Whelan et al., 2007). Therefore, the redox status of the pigmented variants was investigated and compared to the original ancestral isolate. The *phzS* transposon mutant exhibited a similar response to the WT and *mutS* mutant, with a repression of redox in the presence of bile (Figure 5). However, while redox potential in the pigmented variants was comparable to wild-type, *mutS*, and *phzS* transposon mutants in the absence of bile, no reduction in potential was evident upon challenge with bile. This indicates that redox potential is “locked in” in these variants and the metabolic changes that underpin a reduction in redox potential in response to bile may not occur in these strains.

### Akyl Quinolone Signaling Is Central to an Effective Bile Response in *P. aeruginos*a

The observation that pigmented variants no longer exhibited increased biofilm formation or reduced redox potential in the presence of bile led us to further investigate the possible mechanisms involved. Our finding that a *phzS* transposon mutant still retained an intact biofilm response to bile suggested that neither pigment production alone, nor loss of PYO production through mutation of *phzS*, were solely responsible. This raised the intriguing possibility that another signaling system was involved, potentially upstream of PYO, with the AHL-PQS axis considered a likely target. Evidence supporting this hypothesis was provided through SNP analysis whereby LasR was found to exhibit a single amino acid change in both the red and brown mutants from independent replicates of the serial cycling experiment (Supplementary Table 1). Loss of LasR functionality has been previously shown to result in decreased levels of PQS within the cell, with *lasR* mutations described in clinical isolates of *P. aeruginosa* isolated from the lungs of CF patients (Hoffman et al., 2009). Interestingly, the yellow pigmented mutants did not show any change in *lasR* sequence suggesting that mutation of *lasR* occurred after or independent of the emergence of the yellow variant.

**FIGURE 6:**
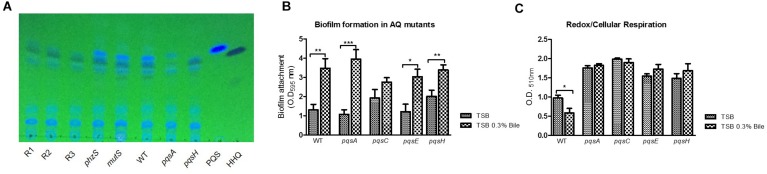
**(A)** TLC analysis indicates loss of PQS production in pigmented mutants, consistent with the possible loss of *lasR* identified in the SNP comparisons. A *phzS* mutant retains PQS production, indicating loss of PQS occurs independent of pigment production. **(B)** Mutation of the biosynthetic genes for PQS did not significantly alter bile induced hyper-biofilm formation. **(C)** Mutation of the biosynthetic genes for PQS resulted in a loss of bile induced redox repression. Data is the mean of at least three independent biological replicates. Statistical analysis was performed by Student’s *t*-test (^∗^*p* ≤ 0.05; ^∗∗^*p* ≤ 0.01; ^∗∗∗^*p* ≤ 0.001).

Analysis of AQ signal and PYO production by Thin Layer Chromatography revealed altered profiles in the red pigmented mutants, consistent with their genotypic profile as putative *lasR* mutants. PQS production was abolished in these strains, while its biological precursor HHQ was retained, comparable to the profile of a *pqsH* mutant (Figure 6A). Interestingly, increased HHQ production was observed in the presence of bile in these strains, indicating that autoinduction by PQS is not required to elicit increased activation of the biosynthetic operon in the presence of bile. It was also notable that both PQS and HHQ production was retained in the *phzS* mutant. As expected, PYO production was also abolished in the red pigmented mutants, unsurprising given the loss of *phzS* activity (data not shown).

From the community perspective, loss of PQS signaling might be expected to result in a less competitive sub-population of *P. aeruginosa*. This is in light of its central role in virulence regulation and its emerging role as an interkingdom effector (Reen et al., 2011; Toyofuku et al., 2017). While loss of PQS would result in the reduction of potentially toxic PYO levels within the cell, this would already be achieved by mutation of the *phzS* gene. Therefore, the role of PQS in response to bile may be a more central mechanism to the adaptation of the global population, within what would appear to be an enhanced biofilm community.

To investigate this, several mutants affected for AQ signaling were tested for their biofilm and redox response to bile. Loss of the PQS biosynthetic genes did not result in the loss of bile induced biofilm formation. This suggested that HHQ and other AQs produced by the *pqsA-E* operon were not required for a functional bile-induced biofilm response (Figure 6B). Interestingly, the loss of the PQS biosynthetic genes resulted in a loss of redox repression in the presence of bile with redox potential considerably higher than the WT counterpart in these mutants (Figure 6C). This finding would strongly implicate a role for alkyl-quinolones in the perturbations of redox potential observed in the presence of bile with the red and brown pigmented derivatives representing bile-adapted redox isolates.

## Discussion/Conclusion

The clinical treatment of respiratory disease is faced with several challenges, not least the rapid decline in novel antibiotic discovery (Cooper and Shlaes, 2011). Innovative approaches to pathogen control have been the focus of intensive research efforts, yet resistance continues to increase globally in spite of improved stewardship and hygiene control (Van Puyvelde et al., 2018). Studies into host factors capable of modulating bacterial behavior are important initiatives facilitating the development of these innovative intervention strategies. There is increasing evidence implicating a role for gastro-oesophageal reflux and subsequent pulmonary aspiration in the progression of CF and other chronic respiratory diseases (van der Doef et al., 2009; Palm et al., 2012; Pauwels et al., 2012; Reen et al., 2014, 2016; Dickson et al., 2017). To date, experimental analysis investigating bile/bacterial interactions has focused on phenotypic responses linked to the acute-chronic switch in *P. aeruginosa* and pathogenesis in general in gastrointestinal pathogens. However, little is known about the consequences for longer term exposure to bile in these organisms and the nature of the adaptation that might occur. Therefore, in this study we performed a growth cycling experiment with *P. aeruginosa* cultured in ASM in the presence and absence of bile, a synthetic media designed to more closely mimic conditions encountered within the sputum rich CF lung. This revealed that global adaptation to bile resulted in enhanced biofilm formation with mutations occurring in signaling systems and *lasR* genes underpinning the emergence of pigmented derivatives.

Phenazine production has been shown to enhance anaerobic survival in *P. aeruginosa* (Glasser et al., 2014), while phenazines have also been shown to influence the physiology of host cells and competing organisms. Production of the phenazine pigment PYO was significantly increased in response to bile in ASM cultures after 24 and 96 h. The increased levels of PYO in the cultures were found to be toxic to the growth of *P. aeruginosa*. This key virulence factor contributes to the pathogenicity of *P. aeruginosa* with significant quantities of this redox-active molecule recovered from the lungs of infected CF patients (Caldwell et al., 2009). The production of PYO is under control of the quorum sensing signaling system and its production involves two distantly encoded biosynthetic operons (*phzA-G*), as well as the modification genes *phzH/M/S.* Several studies have linked production of PYO to redox and virulence processes in *P. aeruginosa* (Dietrich et al., 2006; Price-Whelan et al., 2007; Meirelles and Newman, 2018). PYO has been shown to cause direct damage to human host cells, inhibiting cellular respiration and inducing neutrophil apoptosis (Allen et al., 2005; Manago et al., 2015; Heirali et al., 2016). The importance of PYO was demonstrated in both acute and chronic mouse models, where PYO mutants were less competitive than their wild-type counterparts. PYO is known to play an important role in the establishment of biofilms in *P. aeruginosa*. It is responsible for extracellular DNA release resulting from auto-poisoning and cell death (Das and Manefield, 2012), while also promoting survival of oxidant limited cells within biofilms (Meirelles and Newman, 2018). PYO has also been shown to enhance biofilm formation through second messenger c-di-GMP signaling and its ability to serve as an alternative terminal electron acceptor within the hypoxic biofilm. A recent study by Meirelles et al. has shown that PYO production may have both toxic and beneficial effects (Meirelles and Newman, 2018), analogous in some ways to the role proposed for PQS by Haussler and Becker (2008). PYO-tolerant sub-populations were shown to emerge from nutrient-limited biofilms. In contrast, a susceptible population was sacrificed for eDNA release and structural support in a “net-benefit” arrangement for the population. It is possible therefore that the emergence of pigmented variants that no longer produce PYO following long term exposure to bile, is an adaptation mechanism within the population to the elevated “toxic” levels encountered in the bile positive artificial sputum environment. Elevated toxic levels of PYO may contribute to the selection of a sub-population of PYO defective mutants within the ASM bile microenvironment. Further study is required to elucidate the impact of enhanced pyocyanin production within a bile positive CF lung on the evolutionary trajectory of residing *P. aeruginosa* populations.

The occurrence of pigmented variants within the lungs of patients with respiratory disease has been reported for decades (Meader et al., 1925; Wahba, 1965; Clark et al., 2015; Hocquet et al., 2016; Ketelboeter and Bardy, 2017). Distinction between red and brown pigmented clinical isolates was complicated further by the observation of some isolates that turned from “yellowish to red” (Gessard, 1920). Advances in sequencing and high throughput screening technologies have reinforced the finding that *P. aeruginosa* populations within the lungs of patients with CF are not uniform, but rather display a remarkable level of genotypic and phenotypic heterogeneity (O’Brien et al., 2017). The rationale underpinning the function of these pigmented mutants within lung populations remains to be understood. In our study, while maintained within the growth cycled populations in bile treated ASM, neither the brown nor the red pigmented mutants ever exceeded more than 10% of the population. This would suggest that their function may be crucial in maintaining a PYO positive population of *P. aeruginosa*. Reversion of some colonies following subsequent culturing of red pigmented variants would support this hypothesis. Indeed, the frequency of red pigmented variants in clinical samples is low, ranging from 3.5–6% (Ogunnariwo and Hamilton-Miller, 1975). Brown pyomelanin pigmented variants have been reported at higher frequencies of up to 13% in chronically infected CF patients (Mayer-Hamblett et al., 2014). The selective pressure underpinning the emergence of these pigmented variants in the lungs, urinary tract, and wounds of patients’ remains to be determined. Increased persistence through maintenance of redox-balanced populations within biofilms (Meirelles and Newman, 2018), intraspecific competition through production of pyocins (Hocquet et al., 2016), and tolerance to oxidative stress (Rodriguez-Rojas et al., 2009), may all contribute to the necessity for these pigmented sub-populations.

The quorum sensing AQ signaling system is an effective strategy utilized by *P. aeruginosa* allowing a coordinated gene expression response at the population level. This level of regulation serves as an additional global mechanism of adaptation to external stimuli and has been demonstrated to play a role in both virulence factor production and biofilm formation (Yang et al., 2007; Dubern and Diggle, 2008). The pseudomonas quinolone signal (PQS) has been shown to exert differential effects on individual members of communities of *P. aeruginosa.* It is capable of both sensitizing cells to external stresses and also inducing effective stress responses (Haussler and Becker, 2008). The presence of high levels of PQS has been shown to induce cellular autolysis whilst remaining unaffected cells are triggered to transition to a reduced metabolic and increased PQS tolerant state. The synergy between this selective impact on a population and the recent findings related to the action of PYO on *P. aeruginosa* populations is intriguing and points to a concerted control of populations. As with PYO, PQS represents a central factor in the modulation of population structure and adaptation. The red pigmented derivative was shown to be defective in the production of PQS but not its precursor HHQ, possibly as a consequence of changes in *lasR* sequence. As with the loss of PYO production in a subpopulation of cells, the global reduction of the PQS signaling molecule may represent a successful strategy offsetting any negative effects resulting from over production in a sputum rich environment. The proposed concentration dependent “housekeeping” function of PQS and pyocyanin, whereby both positive and detrimental effects on *P. aeruginosa* communities have been reported are consistent with the emergence of sub-populations in response to extended bile exposure. The molecular mechanisms underlying the emergence of these variants and the collective contribution of each pigmented strain on the persistence of the community as a whole will be the focus of further study to better understand the pathogenesis of *P. aeruginosa* in the lungs of patients with respiratory disease. Furthermore, taking cognizance of the phenotypic and genotypic heterogeneity that exists within *P. aeruginosa* communities in the lung, extension of this study to model (e.g., wild-type PAO1 and PA14) and clinical strains will inform whether the adaptive trajectories described here will also be observed within natural populations.

## Data Availability

The datasets generated for this study can be found in NCBI, PRJNA535434.

## Author Contributions

FR and FO’G conceived the study and supervised the research. SF, FR, and FO’G designed the study. SF performed the laboratory experiments and wrote the manuscript with FR. FO’G provided the critical feedback on the manuscript.

## Conflict of Interest Statement

The authors declare that the research was conducted in the absence of any commercial or financial relationships that could be construed as a potential conflict of interest.

## References

[B1] Allen L. Dockrell D. H. Pattery T. Lee D. G. Cornelis P. Hellewell P. G. (2005). Pyocyanin production by *Pseudomonas aeruginosa* induces neutrophil apoptosis and impairs neutrophil-mediated host defenses *in vivo*. *J. Immunol.* 174 3643–3649. 10.4049/jimmunol.174.6.3643 15749902

[B2] Al-Momani H. Perry A. Stewart C. J. Jones R. Krishnan A. Robertson A. G. (2016). Microbiological profiles of sputum and gastric juice aspirates in cystic fibrosis patients. *Sci. Rep.* 6:26985. 10.1038/srep26985 27245316 PMC4887896

[B3] Blondeau K. Pauwels A. Dupont L. Mertens V. Proesmans M. Orel R. (2010). Characteristics of gastroesophageal reflux and potential risk of gastric content aspiration in children with cystic fibrosis. *J. Pediatr. Gastroenterol. Nutr.* 50 161–166. 10.1097/MPG.0b013e3181acae98 19966579

[B4] Burney P. Jarvis D. Perez-Padilla R. (2015). The global burden of chronic respiratory disease in adults. *Int. J. Tuberc. Lung Dis.* 19 10–20. 10.5588/ijtld.14.0446 25519785

[B5] Cabeen M. T. (2014). Stationary phase-specific virulence factor overproduction by a *lasR* mutant of *Pseudomonas aeruginosa*. *PLoS One* 9:e88743. 10.1371/journal.pone.0088743 24533146 PMC3923063

[B6] Caldwell C. C. Chen Y. Goetzmann H. S. Hao Y. Borchers M. T. Hassett D. J. (2009). *Pseudomonas aeruginosa* exotoxin pyocyanin causes cystic fibrosis airway pathogenesis. *Am. J. Pathol.* 175 2473–2488. 10.2353/ajpath.2009.090166 19893030 PMC2789600

[B7] Caudri D. Turkovic L. Ng J. de Klerk N. H. Rosenow T. Hall G. L. (2018). The association between *Staphylococcus aureus* and subsequent bronchiectasis in children with cystic fibrosis. *J. Cyst. Fibros.* 17 462–469. 10.1016/j.jcf.2017.12.002 29274943

[B8] Clark S. T. Caballero J. D. Cheang M. Coburn B. Wang P. W. Donaldson S. L. (2015). Phenotypic diversity within a *Pseudomonas aeruginosa* population infecting an adult with cystic fibrosis. *Sci. Rep.* 5:10932. 10.1038/srep10932 PMC4456944 26047320

[B9] Cooper M. A. Shlaes D. (2011). Fix the antibiotics pipeline. *Nature* 472:32. 10.1038/472032a 21475175

[B10] Cutting G. R. (2015). Cystic fibrosis genetics: from molecular understanding to clinical application. *Nat. Rev. Genet.* 16 45–56. 10.1038/nrg3849 25404111 PMC4364438

[B11] Darch S. E. McNally A. Harrison F. Corander J. Barr H. L. Paszkiewicz K. (2015). Recombination is a key driver of genomic and phenotypic diversity in a *Pseudomonas aeruginosa* population during cystic fibrosis infection. *Sci. Rep.* 5:7649. 10.1038/srep07649 25578031 PMC4289893

[B12] Das T. Manefield M. (2012). Pyocyanin promotes extracellular DNA release in *Pseudomonas aeruginosa*. *PLoS One* 7:e46718. 10.1371/journal.pone.0046718 23056420 PMC3466280

[B13] Davies E. V. James C. E. Brockhurst M. A. Winstanley C. (2017). Evolutionary diversification of *Pseudomonas aeruginosa* in an artificial sputum model. *BMC Microbiol.* 17:3. 10.1186/s12866-016-0916-z 28056789 PMC5216580

[B14] Dickson R. P. Erb-Downward J. R. Freeman C. M. McCloskey L. Falkowski N. R. Huffnagle G. B. (2017). Bacterial topography of the healthy human lower respiratory tract. *mBio* 8 e2287–e2216. 10.1128/mBio.02287-16 28196961 PMC5312084

[B15] Dietrich L. E. P. Price-Whelan A. Petersen A. Whiteley M. Newman D. K. (2006). The phenazine pyocyanin is a terminal signalling factor in the quorum sensing network of *Pseudomonas aeruginosa*. *Mol. Microbiol.* 61 1308–1321. 10.1111/j.1365-2958.2006.05306.x 16879411

[B16] Douglas T. A. Brennan S. Gard S. Berry L. Gangell C. Stick S. M. (2009). Acquisition and eradication of *P. aeruginosa in young children with cystic fibrosis*. *Eur. Respir. J.* 33 305–311. 10.1183/09031936.00043108 19010992

[B17] Dubern J. F. Diggle S. P. (2008). Quorum sensing by 2-alkyl-4-quinolones in *Pseudomonas aeruginosa* and other bacterial species. *Mol. Biosyst.* 4 882–888. 10.1039/b803796p 18704225

[B18] Feltner J. B. Wolter D. J. Pope C. E. Groleau M. C. Smalley N. E. Greenberg E. P. (2016). LasR variant cystic fibrosis isolates reveal an adaptable quorum-sensing hierarchy in *Pseudomonas aeruginosa*. *mBio* 7:e1513. 10.1128/mBio.01513-16 27703072 PMC5050340

[B19] GBD 2015 Chronic Respiratory Disease Collaborators (2017). Global, regional, and national deaths, prevalence, disability-adjusted life years, and years lived with disability for chronic obstructive pulmonary disease and asthma, 1990-2015: a systematic analysis for the global burden of disease study 2015. *Lancet Respir. Med.* 5 691–706. 10.1016/S2213-2600(17)30293-X 28822787 PMC5573769

[B20] Gessard C. (1920). Technique d’identification des germes pyocyaniques. *Ann. Inst. Pasteur.* 34:88.

[B21] Gibson J. Sood A. Hogan D. A. (2009). *Pseudomonas aeruginosa-Candida albicans* interactions: localization and fungal toxicity of a phenazine derivative. *Appl. Environ. Microbiol.* 75 504–513. 10.1128/AEM.01037-08 19011064 PMC2620721

[B22] Glasser N. R. Kern S. E. Newman D. K. (2014). Phenazine redox cycling enhances anaerobic survival in *Pseudomonas aeruginosa* by facilitating generation of ATP and a proton-motive force. *Mol. Microbiol.* 92 399–412. 10.1111/mmi.12566 24612454 PMC4046897

[B23] Grote J. Krysciak D. Streit W. R. (2015). Phenotypic heterogeneity, a phenomenon that may explain why quorum sensing does not always result in truly homogenous cell behavior. *J. Appl. Environ. Microbiol.* 81 5280–5289. 10.1128/AEM.00900-15 26025903 PMC4510197

[B24] Haussler S. Becker T. (2008). The *pseudomonas* quinolone signal (PQS) balances life and death in *Pseudomonas aeruginosa* populations. *PLoS Pathog.* 4:e1000166. 10.1371/journal.ppat.1000166 18818733 PMC2533401

[B25] Heirali A. McKeon S. Purighalla S. Storey D. G. Rossi L. Costilhes G. (2016). Assessment of the microbial constituents of the home environment of individuals with cystic fibrosis (CF) and their association with lower airways infections. *PLoS One* 11:e0148534. 10.1371/journal.pone.0148534 26859493 PMC4747485

[B26] Hocquet D. Petitjean M. Rohmer L. Valot B. Kulasekara H. D. Bedel E. (2016). Pyomelanin-producing *Pseudomonas aeruginosa* selected during chronic infections have a large chromosomal deletion which confers resistance to pyocins. *Environ. Microbiol.* 18 3482–3493. 10.1111/1462-2920.13336 27119970 PMC5295658

[B27] Hoffman L. R. Kulasekara H. D. Emerson J. Houston L. S. Burns J. L. Ramsey B. W. (2009). *Pseudomonas aeruginosa lasR* mutants are associated with cystic fibrosis lung disease progression. *J. Cyst. Fibros.* 8 66–70. 10.1016/j.jcf.2008.09.006 18974024 PMC2631641

[B28] Jorth P. Staudinger B. J. Wu X. Hisert K. Hayden H. Garudathri J. (2015). Regional isolation drives bacterial diversification within cystic fibrosis lungs. *Cell Host Microbe* 18 307–319. 10.1016/j.chom.2015.07.006 26299432 PMC4589543

[B29] Ketelboeter L. M. Bardy S. L. (2017). Characterization of 2-(2-nitro-4-trifluoromethylbenzoyl)-1,3-cyclohexanedione resistance in pyomelanogenic *Pseudomonas aeruginosa* DKN343. *PLoS One* 12:e0178084. 10.1371/journal.pone.0178084 28570601 PMC5453437

[B30] Liberati N. Urbach J. M. Miyata S. Lee D. G. Drenkard E. Wu G. (2006). An ordered, nonredundant library of *Pseudomonas aeruginosa* strain PA14 transposon insertion mutants. *Proc. Natl. Acad. Sci. U.S.A.* 103 2833–2838. 10.1073/pnas.0511100103 16477005 PMC1413827

[B31] Manago A. Becker K. A. Carpinteiro A. Wilker B. Soddemann M. Seitz A. P. (2015). *Pseudomonas aeruginosa* pyocyanin induces neutrophil death via mitochondrial reactive oxygen species and mitochondrial acid sphingomyelinase. *Antioxid. Redox. Signal.* 22 1097–1110. 10.1089/ars.2014.5979 25686490 PMC4403017

[B32] Mayer-Hamblett N. Rosenfeld M. Gibson R. L. Ramsey B. W. Kulasekara H. D. Retsch-Bogart G. Z. (2014). *Pseudomonas aeruginosa* in vitro phenotypes distinguish cystic fibrosis infection stages and outcomes. *Am. J. Respir. Crit. Care Med.* 190 289–297. 10.1164/rccm.201404-0681OC 24937177 PMC4226041

[B33] Meader P. Robinson G. H. Leonard V. (1925). Pyorubrin, a red water-soluble pigment characteristic of *B. pyocyaneus*. *Am. J. Hyg.* 5:682 10.1093/oxfordjournals.aje.a119690

[B34] Meirelles L. A. Newman D. K. (2018). Both toxic and beneficial effects of pyocyanin contribute to the lifecycle of *Pseudomonas aeruginosa*. *Mol. Microbiol.* 110 995–1010. 10.1111/mmi.14132 30230061 PMC6281804

[B35] Mott L. S. Park J. Murray C. P. Gangell C. L. de Klerk N. H. Robinson P. J. (2012). Progression of early structural lung disease in young children with cystic fibrosis assessed using CT. *Thorax* 67 509–516. 10.1136/thoraxjnl-2011-200912 22201161

[B36] Mowat E. Paterson S. Fothergill J. L. Wright E. A. Ledson M. J. Walshaw M. J. (2011). *Pseudomonas aeruginosa* population diversity and turnover in cystic fibrosis chronic infections. *Am. J. Respir. Crit. Care Med.* 183 1674–1679. 10.1164/rccm.201009-1430OC 21297072

[B37] Muhlebach M. S. Zorn B. T. Esther C. R. Hatch J. E. Murray C. P. Turkovic L. (2018). Initial acquisition and succession of the cystic fibrosis lung microbiome is associated with disease progression in infants and preschool children. *PLoS Pathog.* 14:e1006798. 10.1371/journal.ppat.1006798 29346420 PMC5773228

[B38] O’Brien S. Williams D. Fothergill J. L. Paterson S. Winstanley C. Brockhurst M. A. (2017). High virulence sub-populations in *Pseudomonas aeruginosa* long-term cystic fibrosis airway infections. *BMC Microbiol.* 17:30. 10.1186/s12866-017-0941-6 28158967 PMC5291983

[B39] Ogunnariwo J. Hamilton-Miller J. M. (1975). Brown- and red-pigmented *Pseudomonas aeruginosa*: differentiation between melanin and pyorubrin. *J. Med. Microbiol.* 8 199–203. 10.1099/00222615-8-1-199 805242

[B40] Orlandi V. T. Bolognese F. Chiodaroli L. Tolker-Nielsen T. Barbieri P. (2015). Pigments influence the tolerance of *Pseudomonas aeruginosa* PAO1 to photodynamically induced oxidative stress. *Microbiol* 161 2298–2309. 10.1099/mic.0.000193 26419906

[B41] Palm K. Sawicki G. Rosen R. (2012). The impact of reflux burden on *Pseudomonas* positivity in children with cystic fibrosis. *Pediatr. Pulmonol.* 47 582–587. 10.1002/ppul.21598 22162484

[B42] Pauwels A. Decraene A. Blondeau K. Mertens V. Farre R. Proesmans M. (2012). Bile acids in sputum and increased airway inflammation in patients with cystic fibrosis. *Chest* 141 1568–1574. 10.1378/chest.11-1573 22135379

[B43] Pittman J. E. Calloway E. H. Kiser M. Yeatts J. Davis S. D. Drumm M. L. (2011). Age of *Pseudomonas aeruginosa* acquisition and subsequent severity of cystic fibrosis lung disease. *Ped. Pulmonol.* 46 497–504. 10.1002/ppul.21397 PMC4239995 21194167

[B44] Price-Whelan A. Dietrich L. E. P. Newman D. K. (2007). Pyocyanin alters redox Homeostasis and carbon flux through central metabolic pathways in *Pseudomonas aeruginosa* PA14. *J. Bacteriol.* 189 6372–6381. 10.1128/jb.00505-07 17526704 PMC1951912

[B45] Ramsey K. A. Ranganathan S. Park J. Skoric B. Adams A.-M. Simpson S. J. (2014). Early respiratory infection is associated with reduced spirometry in children with cystic fibrosis. *Am. J. Respir. Crit. Care Med.* 190 1111–1116. 10.1164/rccm.201407-1277OC 25321321

[B46] Reen F. J. Flynn S. Woods D. F. Dunphy N. Chroinin M. N. Mullane D. (2016). Bile signalling promotes chronic respiratory infections and antibiotic tolerance. *Sci. Rep.* 6:29768. 10.1038/srep29768 27432520 PMC4949476

[B47] Reen F. J. Mooij M. J. Holcombe L. J. McSweeney C. M. McGlacken G. P. Morrissey J. P. (2011). The *Pseudomonas* quinolone signal (PQS), and its precursor HHQ, modulate interspecies and interkingdom behaviour. *FEMS Microbiol. Ecol.* 77 413–428. 10.1111/j.1574-6941.2011.01121.x 21539583

[B48] Reen F. J. Woods D. F. Mooij M. J. Adams C. O’Gara F. (2012). Respiratory pathogens adopt a chronic lifestyle in response to bile. *PLoS One* 7:e45978. 10.1371/journal.pone.0045978 23049911 PMC3458808

[B49] Reen F. J. Woods D. F. Mooij M. J. Chroinin M. N. Mullane D. Zhou L. (2014). Aspirated bile: a major host trigger modulating respiratory pathogen colonisation in cystic fibrosis patients. *Eur. J. Clin. Microbiol. Infect. Dis.* 33 1763–1771. 10.1007/s10096-014-2133-8 24816901 PMC4182646

[B50] Rodriguez-Rojas A. Mena A. Martin S. Borrell N. Oliver A. Blazquez J. (2009). Inactivation of the *hmgA* gene of *Pseudomonas aeruginosa* leads to pyomelanin hyperproduction, stress resistance and increased persistence in chronic lung infection. *Microbiology* 155(Pt 4), 1050–1057. 10.1099/mic.0.024745-0 19332807

[B51] Siramulu D. D. (2010). Artificial sputum medium. *Nat. Protoc. Exch.*

[B52] Smania A. M. Segura I. Pezza R. J. Becerra C. Albesa I. Argaraña C. E. (2004). Emergence of phenotypic variants upon mismatch repair disruption in *Pseudomonas aeruginosa*. *Microbiology* 150 1327–1338. 10.1099/mic.0.26751-0 15133095

[B53] Sriramulu D. D. Lünsdorf H. Lam J. S. Römling U. (2005). Microcolony formation: a novel biofilm model of *Pseudomonas aeruginosa* for the cystic fibrosis lung. *J. Med. Microbiol.* 54 667–676. 10.1099/jmm.0.45969-0 15947432

[B54] Stick S. M. Brennan S. Murray C. Douglas T. von Ungern-Sternberg B. S. Garratt L. W. (2009). Bronchiectasis in infants and preschool children diagnosed with cystic fibrosis after newborn screening. *J. Pediatr*. 155 623–628. 10.1016/j.jpeds.2009.05.005 19616787

[B55] Taccetti G. Campana S. Festini F. Mascherini M. Doring G. (2005). Early eradication therapy against *Pseudomonas aeruginosa* in cystic fibrosis patients. *Eur. Respir. J.* 26 458–461. 16135728 10.1183/09031936.05.00009605

[B56] Toyofuku M. Morinaga K. Hashimoto Y. Uhl J. Shimamura H. Inaba H. (2017). Membrane vesicle-mediated bacterial communication. *ISME J.* 11 1504–1509. 10.1038/ismej.2017.13 28282039 PMC5437348

[B57] van der Doef H. P. Arets H. G. Froeling S. P. Westers P. Houwen R. H. (2009). Gastric acid inhibition for fat malabsorption or gastroesophageal reflux disease in cystic fibrosis: longitudinal effect on bacterial colonization and pulmonary function. *J. Pediatr.* 155 629–633. 10.1016/j.jpeds.2009.06.040 19683256

[B58] Van Puyvelde S. Deborggraeve S. Jacobs J. (2018). Why the antibiotic resistance crisis requires a one health approach. *Lancet Infect.* 18 132–134. 10.1016/s1473-3099(17)30704-1 29198739

[B59] Wahba A. H. (1965). Pyorubrin-producing *Pseudomonas aeruginosa*. *Appl. Microbiol.* 13:291. 10.1128/am.13.2.291-292.1965 PMC1058242 14325899

[B60] Wang Y. Gao L. Rao X. Wang J. Yu H. Jiang J. (2018). Characterization of *lasR*-deficient clinical isolates of *Pseudomonas aeruginosa*. *Sci. Rep.* 8:13344. 10.1038/s41598-018-30813-y 30190495 PMC6127196

[B61] Wassermann T. Meinike Jorgensen K. Ivanyshyn K. Bjarnsholt T. Khademi S. M. Jelsbak L. (2016). The phenotypic evolution of *Pseudomonas aeruginosa* populations changes in the presence of subinhibitory concentrations of ciprofloxacin. *Microbiology* 162 865–875. 10.1099/mic.0.000273 26953154

[B62] Williams D. Evans B. Haldenby S. Walshaw M. J. Brockhurst M. A. Winstanley C. (2015). Divergent, coexisting *Pseudomonas aeruginosa* lineages in chronic cystic fibrosis lung infections. *Am. J. Respir. Crit. Care Med.* 191 775–785. 10.1164/rccm.201409-1646OC 25590983 PMC4407486

[B63] Workentine M. L. Sibley C. D. Glezerson B. Purighalla S. Norgaard-Gron J. C. Parkins M. D. (2013). Phenotypic heterogeneity of *Pseudomonas aeruginosa* populations in a cystic fibrosis patient. *PLoS One* 8:e60225. 10.1371/journal.pone.0060225 23573242 PMC3616088

[B64] Yang L. Barken K. B. Skindersoe M. E. Christensen A. B. Givskov M. Tolker-Nielsen T. (2007). Effects of iron on DNA release and biofilm development by *Pseudomonas aeruginosa*. *Microbiology* 153(Pt 5), 1318–1328. 10.1099/mic.0.2006/004911-0 17464046

[B65] Zecca E. De Luca D. Baroni S. Vento G. Tiberi E. Romagnoli C. (2008). Bile acid-induced lung injury in newborn infants: a bronchoalveolar lavage fluid study. *Pediatrics* 121 e146–e149. 10.1542/peds.2007-1220 18166532

