# Supplementary material for: Exposure to Bile Leads to the Emergence of Adaptive Signaling Variants in the Opportunistic Pathogen Pseudomonas aeruginosa
Source: Front Microbiol. 2019 Aug 29;10:2013. doi: 10.3389/fmicb.2019.02013 (PMC6727882; doi:10.3389/fmicb.2019.02013)
Supplement: Supplementary file 2 [file Data_Sheet_1.PDF]

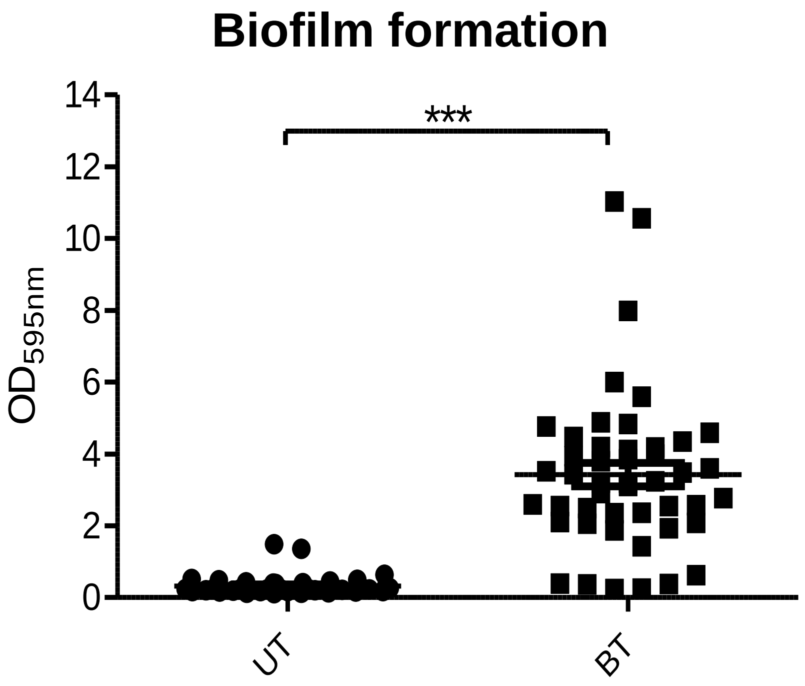

Supplementary Figure 1; Biofilm formation in 48 isolates from untreated ASM versus 48 strains isolates from ASM supplemented with bile. Biofilm is significantly higher in strains isolates from ASM supplemented with bile. Data is the mean of at least three independent biological replicates. Statistical analysis was performed by Student's t-test (***$p \leq 0.001$).
