# Supplementary material for: Exposure to Bile Leads to the Emergence of Adaptive Signaling Variants in the Opportunistic Pathogen Pseudomonas aeruginosa
Source: Front Microbiol. 2019 Aug 29;10:2013. doi: 10.3389/fmicb.2019.02013 (PMC6727882; doi:10.3389/fmicb.2019.02013)
Supplement: Supplementary file 3 [file Data_Sheet_2.PDF]

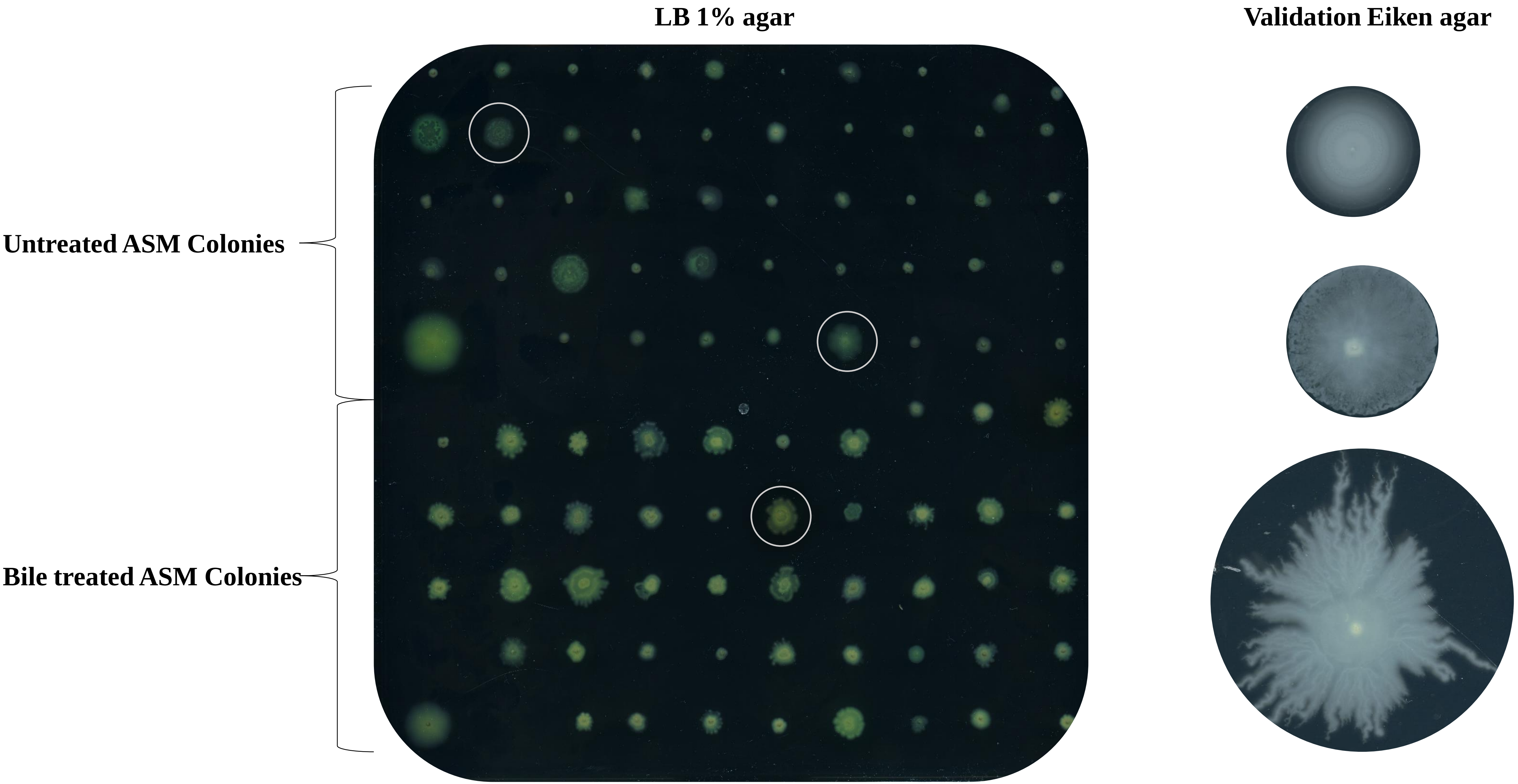

Supplementary Figure 2; Swarming Motility of 48 strains isolated from untreated ASM and 48 strains isolated from ASM supplemented with bile on LB 1% agar with a representative validation of colonies on Eiken agar. Colonies isolated from ASM supplemented with bile retained their ability swarm while those isolated from untreated ASM appeared to become swarming deficient.
