# Supplementary material for: Exposure to Bile Leads to the Emergence of Adaptive Signaling Variants in the Opportunistic Pathogen Pseudomonas aeruginosa
Source: Front Microbiol. 2019 Aug 29;10:2013. doi: 10.3389/fmicb.2019.02013 (PMC6727882; doi:10.3389/fmicb.2019.02013)
Supplement: Supplementary file 4 [file Data_Sheet_3.PDF]

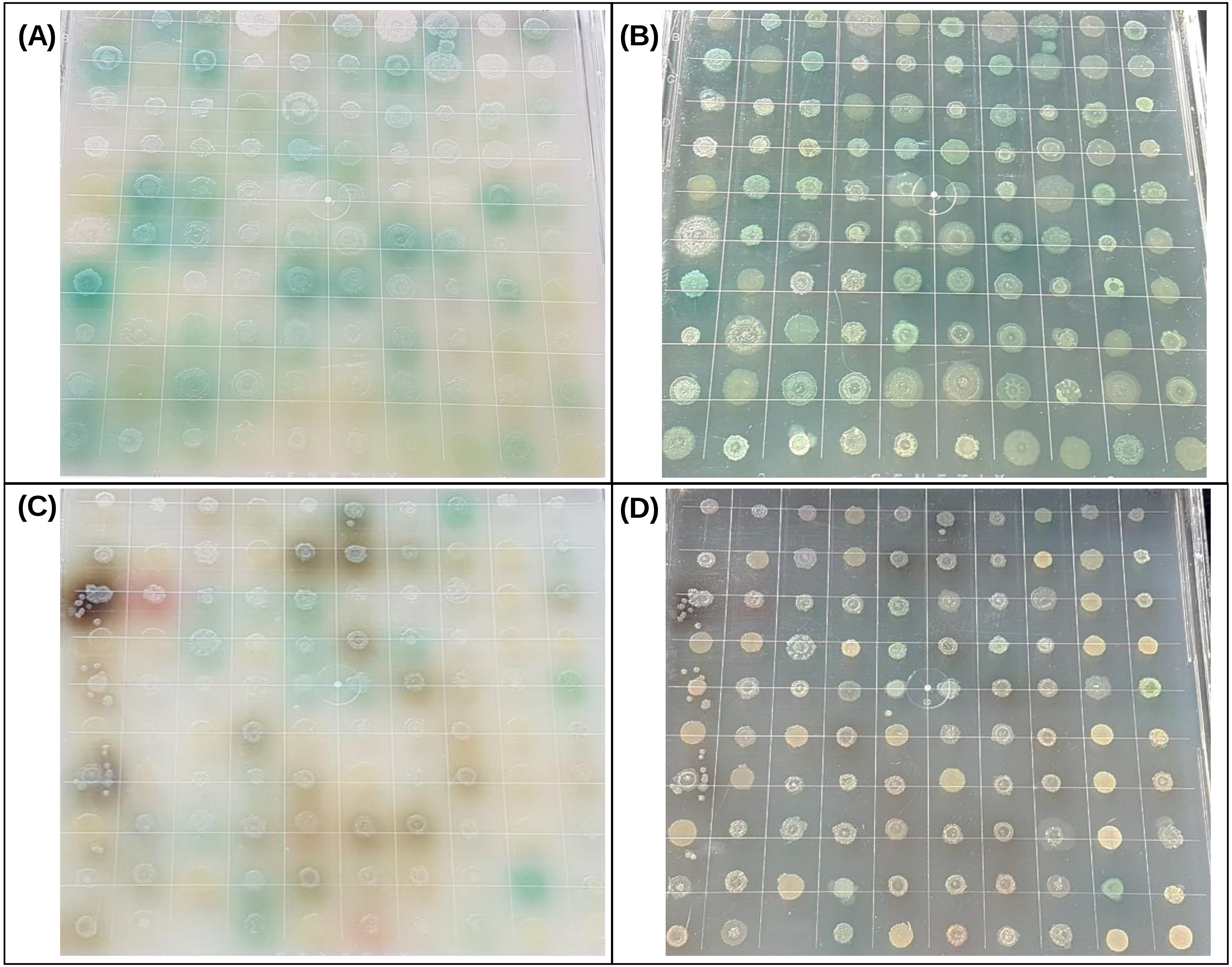

Supplementary Figure 3; Colony morphology analysis on LBA of (A&B) 100 randomly isolated untreated ASM colonies and (C&D) 100 randomly isolates ASM supplemented with bile colonies. A and C are surface views of the plate while B and D are under side views of the same plate as pictured.
