# Supplementary material for: Exposure to Bile Leads to the Emergence of Adaptive Signaling Variants in the Opportunistic Pathogen Pseudomonas aeruginosa
Source: Front Microbiol. 2019 Aug 29;10:2013. doi: 10.3389/fmicb.2019.02013 (PMC6727882; doi:10.3389/fmicb.2019.02013)
Supplement: Supplementary file 5 [file Data_Sheet_4.PDF]

(A)

Gene Number	Gene Name	Gene Function
PA14_00970		Hypothetical
PA14_08120		Tail length determinator protein
PA14_11730		Hypothetical
PA14_14400		Hypothetical
PA14_14530		Hypothetical
PA14_52190	*rumA*	23S rRNA-methyluridine methyltransferase
PA14_56100		Hypothetical
PA14_59200		Hypothetical
PA14_59980		Hypothetical
PA14_61050	*mscL*	Large-conductance mechanosensitive channel
PA14_68020		Hypothetical
PA14_14570		tRNA-leucine

(B)

Gene Number	Gene Name	Gene Function
PA14_17660		Hypothetical
PA14_25030 *		Hypothetical
PA14_28830		Hypothetical
PA14_30440		Hypothetical
PA14_33200		Hypothetical
PA14_34870	*chiC*	Chitinase
PA14_35290 *	*gnd*	Gluconate dehydrogenase
PA14_37680		Hypothetical
PA14_40020		Hypothetical
PA14_41560		Assimilatory nitrate reductase
PA14_42220		Sensor domain containing protein
PA14_46660		RNA polymerase ECF subfamily sigma 70 factor
PA14_53980		Hypothetical
PA14_69040		5-Formyltetrahydrofolate cyclo-ligase
PA14_16190		Hypothetical

(C)

Gene Number	Gene Name	Gene Function
PA14_00980 *	*fha1*	Type VI secretion
PA14_11100 *	*cupB5*	Adhesive protein
PA14_15200 *		Hypothetical
PA14_23460	*orfN*	Group 4 glycosyl transferase
PA14_29520		Type II secretion
PA14_42600	*pscP*	Translocation protein in type III secretion
PA14_52250		Two component response regulator
PA14_54750		Hypothetical
PA14_69760		Fimbrial protein
PA14_70580 *		Hypothetical

(D)

Gene Number	Gene Name	Gene Function
PA14_01970		RND efflux transporter
PA14_04440		Hypothetical
PA14_05300		TonB domain containing protein
PA14_09400	*phzS*	Hypothetical
PA14_12420		
PA14_37900 *	*sppR*	TonB dependent receptor
PA14_46660 *		RNAP ECF subfamily σ70 factor
PA14_44300 *	*aer*	Aerotaxis receptor

Supplementary Figure 4; Insertion/Deletions and premature stop codons (highlighted in grey) in coding regions in (A) All three pigmented derivatives (B) Brown pigmented derivative (C) Yellow pigmented derivative and (D) Red pigmented derivative.
