# Supplementary material for: Exposure to Bile Leads to the Emergence of Adaptive Signaling Variants in the Opportunistic Pathogen Pseudomonas aeruginosa
Source: Front Microbiol. 2019 Aug 29;10:2013. doi: 10.3389/fmicb.2019.02013 (PMC6727882; doi:10.3389/fmicb.2019.02013)
Supplement: Supplementary file 6 [file Data_Sheet_5.PDF]

Supplementary Figure 5; PCR sequence analysis of *phzS* and *hmgA* in three independent red pigmented isolates and three independent brown pigmented isolates.

phzS

Red isolate 1

```
PA14      ATGAGCGAACCCATCGATATCCTCATTGCCGGCGCCGGCATCGGCGGCCTCAGCTGCGCC 60
F1        ---------------------------------------------------------GCC 3
F2        ---------------------------------------------------------GCC 3
                                                                   ***

PA14      CTGGCCCTGCACCAGGCCGGCATCGGCAAGGTCACGCTGCTGGAAAGCAGCAGCGAGATA 120
F1        CTGGCCCTGCACCAGGCCGGCATCGGCAAGGTCACGCTGCTGGAAAGCAGCAGCGAGATA 63
F2        CTGGCCCTGCACCAGGCCGGCATCGGCAAGGTCACGCTGCTGGAAAGCAGCAGCGAGATA 63
          ************************************************************

PA14      CGCC-CCCTTGGCGTCGGCATCAATATCCAGCCGGCGGCGGTCGAGGCCCTCGCCGAACT 179
F1        CGCCCCCCTTGGCGTCGGCATCAATATCCAGCCGGCGGCGGTCGAGGCCCTCGCCGAACT 123
F2        CGCCCCCCTTGGCGTCGGCATCAATATCCAGCCGGCGGCGGTCGAGGCCCTCGCCGAACT 123
          **** *******************************************************

PA14      GGGCCTCGGCCCGGCGCTGGCAGCCACCGCCATCCCCACCCATGAGCTGCGCTACATCGA 239
F1        GGGCCTCGGCCCGGCGCTGGCAGCCACCGCCATCCCCACCCATGAGCTGCGCTACATCGA 183
F2        GGGCCTCGGCCCGGCGCTGGCAGCCACCGCCATCCCCACCCATGAGCTGCGCTACATCGA 183
          ************************************************************

PA14      CCAGAGCGGCGCCACGGTATGGTCCGAGCCGCGCGGGGTGGAAGCCGGCAACGCCTATCC 299
F1        CCAGAGCGGCGCCACGGTATGGTCCGAGCCGCGCGGGGTGGAAGCCGGCAACGCCTATCC 243
F2        CCAGAGCGGCGCCACGGTATGCTCCGAGCCGCGCGGGGTGGAAGCCGGCAACGCCTATCC 243
          ********************* **************************************

PA14      GCAGTACTCGATCCATCGCGGCGAACTGCAGATGATCCTGCTCGCCGCGGTGCGCGAGCG 359
F1        GCAGTACTCGATCCATCGCGGCGATCTGCAGATGATCCTGCTCGCCGCGGTGCGCGAGCG 303
F2        GCAGTACTCGATCCATCGCGGCGAACTGCAGATGATCCTGCTCGCCGCGGTGCGCGAGCG 303
          ************************ ***********************************

PA14      TCTCGGCCAACAGGCGGTACGCACCGGTCTCGGCGTGGAGCGCATCGAAGAGCGCGACGG 419
F1        TCTCGGCCAACAGGCGGTACGCACCGGTCTCGGCGTGGAGCGCATCGAAAAGCGCGACGG 363
F2        TCTCGGCCAACAGGCGGTACGCACCGGTCTCGGCGTGGAGCGCATCGAAGAGCGCGACGG 363
          ************************************************* **********

PA14      CCGCGTACTGATCGGCGCCCGCGACGGACACGGCAAGCCCCTGGCGCTCGGTGCCGATGT 479
F1        CCGCGTACTGATCGGCGCCCGCGACGGACACGGCAAGCCCCTGGCGCTCGGTGCCGATGT 423
F2        CCGCGTACTGATCGGCGCCCGCGACGGACACGGCAAGCCCCTGGCGCTCGGTGCCGATGT 423
          ************************************************************

PA14      GCTGGTCGGCGCCGACGGCATCCATTCGGCGGTCCGCGCGCACCTGCATCCCGACCAGGG 539
F1        GCTGGTCGGCGCCGACGGCATCCATTCGGCGGTCCGCGCGCACCTGCATCCCGACCAGGG 483
F2        GCTGGTCGGCGCCGACGGCATCCATTCGGCGGTCCGCGCGCACCTGCATCCCGACCAGGG 483
          ************************************************************

PA14      GCCGCTGTCCCACGGTGGGATCACCATGTGGCGCGGCGTCACCGAGTTCGACCGCTTCCT 599
F1        GCCG-------------------------------------------------------- 487
F2        GCCG-------------------------------------------------------- 487
```

```
                    ****
```

Red isolate 2

```
PA14      ------------------------------------------------------------ 0
F1        CTCGGAGCCGGGCCGTAGGGCGAATAACGCCACCGGCGTTATCCGCCGCTGCGCCGACGT 60
F2        CTCGGAGCCGGGCCGTAGGGCGAATAACGCCACCGGCGTTATCCGCCGCTGCGCCGACGT 60


PA14      ------------------------------------------------------------ 0
F1        TTCATCGCGGTAGACGGTCATCCGCCCCAGCCGAACACCCATCGATTCGAACACTCGAGA 120
F2        TTCATCGCGGTAGACGGTCATCCGCCCCAGCCGAACACCCATCGATTCGAACACTCGAGA 120


PA14      -------------ATGAGCGAACCCATCGATATCCTCATTGCCGGCGCCGGCATCGGCGG 47
F1        AAAGGAAGCACCCATGAGCGAACCCATCGATATCCTCATTGCCGGCGCCGGCATCGGCGG 180
F2        AAAGGAAGCACCCATGAGCGAACCCATCGATATCCTCATTGCCGGCGCCGGCATCGGCGG 180
                       ***********************************************

PA14      CCTCAGCTGCGCCCTGGCCCTGCACCAGGCCGGCATCGGCAAGGTCACGCTGCTGGAAAG 107
F1        CCTCAGCTGCGCCCTGGCCCTGCACCAGGCCGGCATCGGCAAGGTCACGCTGCTGGAAAG 240
F2        CCTCAGCTGCGCCCTGGCCCTGCACCAGGCCCGCATCGGCAAGGTCACGCTGCTGGAAAG 240
          ******************************* ****************************

PA14      CAGCAGCGAGATACGCCC-CCTTGGCGTCGGCATCAATATCCAGCCGGCGGCGGTCGAGG 166
F1        CAGCAGCGAGATACGCCCCCCTTGGCGTCGGCATCAATATCCAGCCGGCGGCGGTCGAGG 300
F2        CAGCAGCGAGATACGCCCCCCTTGGCGTCGGCATCAATATCCAGCCGGCGGCGGTCGAGG 300
          ****************** *****************************************

PA14      CCCTCGCCGAACTGGGCCTCGGCCCGGCGCTGGCAGCCACCGCCATCCCCACCCATGAGC 226
F1        CCCTCGCCGAACTGGGCCTCGGCCCGGCGCTGGCAGCCACCGCCATCCCCACCCATGAGC 360
F2        CCCTCGCCGAACTGGGCCTCGGCCCGGCGCTGGCAGCCACCGCCATCCCCACCCATGAGC 360
          ************************************************************

PA14      TGCGCTACATCGACCAGAGCGGCGCCACGGTATGGTCCGAGCCGCGCGGGGTGGAAGCCG 286
F1        TGCGCTACATCGACCAGAGCGGCGCCACGGTATGGTCCGAGCCGCGCGGGGTGGAAGCCG 420
F2        TGCGCTACATCGACCAGAGCGGCGCCACGGTATGGTCCGAGCCGCGCGGGGTGGAAGCCG 420
          ************************************************************

PA14      GCAACGCCTATCCGCAGTACTCGATCCATCGCGGCGAACTGCAGATGATCCTGCTCGCCG 346
F1        GCAACGCCTATCCGCAGTACTCGATCCATCGCGGCGAACTGCAGATGATCCTGCTCGCCG 480
F2        GCAACGCCTATCCGCAGTACTCGATCCATCGCGGCGAACTGCAGATGATCCTGCTCGCCG 480
          ************************************************************

PA14      CGGTGCGCGAGCGTCTCGGCCAACAGGCGGTACGCACCGGTCTCGGCGTGGAGCGCATCG 406
F1        CGGTGCGCGAGCGTCTCGGCCAACAGGCGGTACGCACCGGTCTCGGCGTGGAGCGCATCG 540
F2        CGGTGCGCGAGCGTCTCGGCCAACAGGCGGTACGCACCGGTCTCGGCGTGGAGCGCATCG 540
          ************************************************************

PA14      AAGAGCGCGACGGCCGCGTACTGATCGGCGCCCGCGACGGACACGGCAAGCCCCTGGCGC 466
F1        AAGAGCGCGACGGCCGCGTACTGATCGGCGCCCGCGACGGACACGGCAAGCCCCTGGCGC 600
F2        AAGAGCGCGACGGCCGCGTACTGATCGGCGCCCGCGACGGACACGGCAAGCCCCTGGCGC 600
          ************************************************************

PA14      TCGGTGCCGATGTGCTGGTCGGCGCCGACGGCATCCATTCGGCGGTCCGCGCGCACCTGC 526
F1        TCGGTGCCGATGTGCTGGTCGGCGCCGACGGCATCCATTCGGCGGTCCGCGCGCACCTGC 660
```

```
F2         TCGGTGCCGATGTGCTGGTCGGCGCCGACGGCATCCATTCGGCGGTCCGCGCGCACCTGC 660
           ************************************************************

PA14       ATCCCGACCAGGGGCCGCTGTCCCACGGTGGGATCACCATGTGGCGCGGCGTCACCGAGT 586
F1         ATCCCGACCAGGGGCCGCTGTCCCACGGTGGGATCACCATGTGGCGCGGCGTCACCGAGT 720
F2         ATCCCGACCAGGGGCCGCTGTCCCACGGTGGGATCACCATGTGGCGCGGCGTCACCGAGT 720
           ************************************************************

PA14       TCGACCGCTTCCTCGACGGCAAGACCATGATCGTCGCCAACGACGAGCACTGGTCGCGCC 646
F1         TCGACCGCTTCCTCGACGGCAAGACCATGATCGTCGCCAACGACGAGCACTGGTCGCGCC 780
F2         TCGACCGCTTCCTCGACGGCAAGACCTTGATCGTCGCCAACGACGAGCACTGGTCGCGCC 780
           ************************** *********************************

PA14       TGGTCGCCTATCCGATCTCGGCGCGCCACGCGGCCGAAGGCAAGTCGCTGGTGAACTGGG 706
F1         TGGTCGCCTATCCGATCTCGGCGCGCCACGCGGCCGAAGGCAAGTCGCTGGTGAACTGGG 840
F2         TGGTCGCCTATCCGATCTCGGCGCGCCACGCGGCCGAAGGCAAGTCGCTGGTGAACTGGG 840
           ************************************************************

PA14       TGTGCATGGTGCCGAGCGCCGCCGTCGGCCAGCTCGACAACGAGGCCGACTGGAACCGCA 766
F1         TGTGCATGGTGCCAAGCGCCGCCGTCGGCCAGCTCGACAACGAGGCCGACTGGAACCGCA 900
F2         TGTGCATGGTGCCAAGCGCCGCCGTCGGCCAGCTCGACAACGAGGCCGACTGGAACCGCA 900
           ************* **********************************************

PA14       ACGGACGCCTGGAAGACGTGTTGCCGTTCTTCGCCGACTGGGACCTGGGCTGGTTCGACA 826
F1         ACGGACGCCTGGAAGACGTGTTGCCGTTCTTCGCCGACTGGGACCTGGGCTGGTTCGACA 960
F2         ACGGACGCCTGGAAGACGTGTTGCCGTTCTTCGCCGACTGGGACCTGGGCTGGTTCGACA 960
           ************************************************************

PA14       TCCGCGACCTGCTGACCCGCAACCAGTTGATCCTGCAGTACCCGATGGTCGACCGCGATC 886
F1         TCCGCGACCTGCTGACCCGCAACCAGTTGATCCTGCAGTACCCGATGGTCGACCGCG--- 1017
F2         TCCGCGACCTGCTGACCCGCAACCAGTTGATCCTGCAGTACCCGATGGTCGACCGCG--- 1017
           *********************************************************
```

Red isolate 3

```
PA14       ------------ATGAGCGAACCCATCGATATCCTCATTGCCGGCGCCGGCATCGGCGGC 48
F1         AAGGAAGCACCCATGAGCGAACCCATCGATATCCTCATTGCCGGCGCCGGCATCGGCGGC 60
F2         AAGGAAGCACCCATGAGCGAACCCATCGATATCCTCATTGCCGGCGCCGGCATCGGCGGC 60
                       ************************************************

PA14       CTCAGCTGCGCCCTGGCCCTGCACCAGGCCGGCATCGGCAAGGTCACGCTGCTGGAAAGC 108
F1         CTCAGCTGCGCCCTGGCCCTGCACCAGGCCGGCATCGGCAAGGTCACGCTGCTGGAAAGC 120
F2         CTCAGCTGCGCCCTGGCCCTGCACCAGGCCGGCATCGGCAAGGTCACGCTGCTGGAAAGC 120
           ************************************************************

PA14       AGCAGCGAGATACGCCC-CCTTGGCGTCGGCATCAATATCCAGCCGGCGGCGGTCGAGGC 167
F1         AGCAGCGAGATACGCCCCCCTTGGCGTCGGCATCAATATCCAGCCGGCGGCGGTCGAGGC 180
F2         AGCAGCGAGATACGCCCCCCTTGGCGTCGGCATCAATATCCAGCCGGCGGCGGTCGAGGC 180
           ***************** ******************************************

PA14       CCTCGCCGAACTGGGCCTCGGCCCGGCGCTGGCAGCCACCGCCATCCCCACCCATGAGCT 227
F1         CCTCGCCGAACTGGGCCTCGGCCCGGCGCTGGCAGCCACCGCCATCCCCACCCATGAGCT 240
F2         CCTCGCCGAACTGGGCCTCGGCCCGGCGCTGGCAGCCACCGCCATCCCCACCCATGAGCT 240
           ************************************************************

PA14       GCGCTACATCGACCAGAGCGGCGCCACGGTATGGTCCGAGCCGCGCGGGGTGGAAGCCGG 287
F1         GCGCTACATCGACCAGAGCGGCGCCACGGTATGGTCCGAGCCGCGCGGGGTGGAAGCCGG 300
```

```
F2          GCGCTACATCGACCAGAGCGGCGCCACGGTATGGTCCGAGCCGCGCGGGGTGGAAGCCGG 300
            ************************************************************

PA14        CAACGCCTATCCGCAGTACTCGATCCATCGCGGCGAACTGCAGATGATCCTGCTCGCCGC 347
F1          CAACGCCTATCCGCAGTACTCGATCCATCGCGGCGAACTGCAGATGATCCTGCTCGCCGC 360
F2          CAACGCCTATCCGCAGTACTCGTTCCATCGCGGCGAACTGCAGATGATCCTGCTCGCCGC 360
            ********************** *************************************

PA14        GGTGCGCGAGCGTCTCGGCCAACAGGCGGTACGCACCGGTCTCGGCGTGGAGCGCATCGA 407
F1          GGTGCGCGAGCGTCTCGGCCAACAGGCGGTACGCACCGGTCTCGGCGTGGAGCGCATCGA 420
F2          GGTGCGCGAGCGTCTCGGCCAACAGGCGGTACGCACCGGTCTCGGCGTGGAGCGCATCGA 420
            ************************************************************

PA14        AGAGCGCGACGGCCGCGTACTGATCGGCGCCCGCGACGGACACGGCAAGCCCCTGGCGCT 467
F1          AGAGCGCGACGGCCGCGTACTGACCGGCGCCCGCGACGGACACGGCAAGCCCCTGGCGCT 480
F2          AGAGCGCGACGGCCGCGTACTGATCGGCGCCCGCGACGGACACGGCAAGCCCCTGGCGCT 480
            *********************** ************************************

PA14        CGGTGCCGATGTGCTGGTCGGCGCCGACGGCATCCATTCGGCGGTCCGCGCGCACCTGCA 527
F1          CGGTGCCGATGTGCTGGTCGGCGCCGACGGCATCCATTCGGCGGTCCGCGCGCACCTGCA 540
F2          CGGTGCCGATGTGCTGGTCGGCGCCGACGGCATCCATTCGGCGGTCCGCGCGCACCTGCA 540
            ************************************************************

PA14        TCCCGACCAGGGGCCGCTGTCCCACGGTGGGATCACCATGTGGCGCGGCGTCACCGAGTT 587
F1          TCCCGACCAGGGGCCGCTGTCCCACGGTGGGATCACCATGTGGCGCGGCGTCACCGAGTT 600
F2          TCCCGACCAGGGGCCGCTGTCCCACGGTGGGATCACCATGTGGCGCGGCGTCACCGAGTT 600
            ************************************************************

PA14        CGACCGCTTCCTCGACGGCAAGACCATGATCGTCGCCAACGACGAGCACTGGTCGCGCCT 647
F1          CGACCGCTTCCTCGACGGCAAGACCATGATCGTCGCCAACGACGAGCACTGGTCGCGCCT 660
F2          CGACCGCTTCCTCGACGGCAAGACCATGATCGTCGCCAACGACGAGCACTGGTCGCGCCT 660
            ************************************************************

PA14        GGTCGCCTATCCGATCTCGGCGCGCCACGCGGCCGAAGGCAAGTCGCTGGTGAACTGGGT 707
F1          GGTCGCCTATCCGATCTCGGCGCGCCACGCGGCCGAAGGCAAGTCGCTGGTGAACTGGGT 720
F2          GGTCGCCTATCCGATCTCGGCGCGCCACGCGGCCGAAGGCAAGTCGCTGGTGAACTGGGT 720
            ************************************************************

PA14        GTGCATGGTGCCGAGCGCCGCCGTCGGCCAGCTCGACAACGAGGCCGACTGGAACCGCAA 767
F1          GTGCATGGTGCCGAGCGCCGCCGTCGGCCAGCTCGACAACGAGGCCGACTGGAACCGCAA 780
F2          GTGCATGGTGCCAAGCGCCGCCGTCGGCCAGCTCGACAACGAGGCCGACTGGAACCGCAA 780
            ************ ***********************************************

PA14        CGGACGCCTGGAAGACGTGTTGCCGTTCTTCGCCGACTGGGACCTGGGCTGGTTCGACAT 827
F1          CGGACGCCTGGAAGACGTGTTGCCGTTCTTCGCCGACTGGGACCTGGGCTGGTTCGACAT 840
F2          CGGACGCCTGGAAGACGTGTTGCCGTTCTTCGCCGACTGGGACCTGGGCTGGTTCGACAT 840
            ************************************************************

PA14        CCGCGACCTGCTGACCCGCAACCAGTTGATCCTGCAGTACCCGATGGTCGACCGCGATCC 887
F1          CCGCGACCTGCTGACCCGCAACCAGTTGATCCTGCAGTACCCGATGGTCGACCGCAAT-- 898
F2          CCGCGACCTGCTGACCCGCAACCAGTTGATCCTGCAGTACCCGATGGTCGACCGCAAT-- 898
            ******************************************************* **
```

hmgA

Brown isolate 1

```
F       GCTGCCCCACTCCCCGGAGGCCTCAGATGAACCTCGACTCCACTGCCCTCGCCTATCAAT 60
PA14    --------------------------ATGAACCTCGACTCCACTGCCCTCGCCTATCAAT 34
R       ------------------------------------------------------------ 0

F       CGGGCTTCGGCAACGAATTCAGCAGCGAAGCGCTCCCCGGCGCCCTGCCGGTCGGCCAGA 120
PA14    CGGGCTTCGGCAACGAATTCAGCAGCGAAGCGCTCCCCGGCGCCCTGCCGGTCGGCCAGA 94
R       ------------------------------------------------------------0

F       ACTCCCCGCAGAAAGCGCCCTACGGCCTGTACGCCGAACTGCTCTCCGGCACCGCCTTCA 180
PA14    ACTCCCCGCAGAAAGCGCCCTACGGCCTGTACGCCGAACTGCTCTCCGGCACCGCCTTCA 154
R       ------------------------------------------------------------ 0

F       CCATGGCTCGCAGCGAGGCCCGGCGCACCTGGCTATACCGCATCACGCCGTCGGCCAAGC 240
PA14    CCATGGCTCGCAGCGAGGCCCGGCGCACCTGGCTATACCGCATCACGCCGTCGGCCAAGC 214
R       ------------------------------------------------------------ 0

F       ATCCGCCGTTCCGCCGCCTGGAACGACAGATCGCCGGTGCCGAACTGGATGCGCCGACCC 300
PA14    ATCCGCCGTTCCGCCGCCTGGAACGACAGATCGCCGGTGCCGAACTGGATGCGCCGACCC 274
R       ------------------------------------------------------------ 0

F       CCAACCGCCTGCGCTGGGACCCGCTGGCACTGCCCGAGCAGCCCACCGACTTCCTCGACG 360
PA14    CCAACCGCCTGCGCTGGGACCCGCTGGCACTGCCCGAGCAGCCCACCGACTTCCTCGACG 334
R       ------------------------------------------------------------ 0

F       GCCTGCTGCGCATGGCCGCCAACGCGCCCGGCGACAAGCCCGCCGGCGTGAGCATCTACC 420
PA14    GCCTGCTGCGCATGGCCGCCAACGCGCCCGGCGACAAGCCCGCCGGCGTGAGCATCTACC 394
R       ------------------------------------------------------------ 0

F       AGTACCTGGCCAACCGCTCGATGGAGCGTTGCTTCTACGACGCCGACGGCGAACTGCTGC 480
PA14    AGTACCTGGCCAACCGCTCGATGGAGCGTTGCTTCTACGACGCCGACGGCGAACTGCTGC 454
R       ---------------------------------------------ACGGCGAACTGCTGC 15
                                                     ***************

F       TGGTCCCGCAGTTGGGCCGCCTGCGCCTGTGCACCGAACTCGGCGCGCTGCAGGTCGAAC 540
PA14    TGGTCCCGCAGTTGGGCCGCCTGCGCCTGTGCACCGAACTCGGCGCGCTGCAGGTCGAAC 514
R       TGGTCCCGCAGTTGGGCCGCCTGCGCCTGTGCACCGAACTCGGCGCGCTGCAGGTCGAAC 75
        ************************************************************

F       CGCTGGAGATCGCGGTGATCCCGCGCGGGATGAAGTTCCGCGTCGAGCTGCTCGACGGCG 600
PA14    CGCTGGAGATCGCGGTGATCCCGCGCGGGATGAAGTTCCGCGTCGAGCTGCTCGACGGCG 574
R       CGCTGGAGATCGCGGTGATCCCGCGCGGGATGAAGTTCCGCGTCGAGCTGCTCGACGGCG 135
        ************************************************************

F       AGGCACGCGGCTATATCGCCGAGAACCACGGCGCGCCGCTGCGCCTGCCCGACCTCGGCC 660
PA14    AGGCACGCGGCTATATCGCCGAGAACCACGGCGCGCCGCTGCGCCTGCCCGACCTCGGCC 634
R       AGGCACGCGGCTATATCGCCGAGAACCACGGCGCGCCGCTGCGCCTGCCCGACCTCGGCC 195
        ************************************************************

F       CGATCGGCAGCAATGGCCTGGCCAATCCGCGCGACTTCCTGGCCCCCGGTGGCGCGCTAC 720
PA14    CGATCGGCAGCAATGGCCTGGCCAATCCGCGCGACTTCCTGGCC-CCGGTGGCGCGCTAC 693
```

```
R          CGATCGGCAGCAATGGCCTGGCCAATCCGCGCGACTTCCTGGCC-CCGGTGGCGCGCTAC 254
           ******************************************** ***************

F          GAAGACAGCCGCCAGCCGCTGCAACTGGTGCAGAAATACCTCGGCGAGCTGTGGGCCTGC 780
PA14       GAAGACAGCCGCCAGCCGCTGCAACTGGTGCAGAAATACCTCGGCGAGCTGTGGGCCTGC 753
R          GAAGACAGCCGCCAGCCGCTGCAACTGGTGCAGAAATACCTCGGCGAGCTGTGGGCCTGC 314
           ************************************************************

F          GAGCTTGACCACTCGCCGCTTGGACG---------------------------------- 806
PA14       GAGCTTGACCACTCGCCGCTGGACGTGGTCGCCTGGCACGGCAACAACGTGCCCTACAAG 813
R          GAGCTTGACCACTCGCCGCTGGACGTGGTCGCCTGGCACGGCAACAACGTGCCCTACAAG 374
           ******************** *

F          ------------------------------------------------------------ 806
PA14       TACGACCTGCGCCGCTTCAACACCATCGGCACGGTCAGCTTCGACCACCCGGACCCGTCG 873
R          TACGACCTGCGCCGCTTCAACACCATCGGCACGGTCAGCTTCGACCACCCGGACCCGTCG 434

F          ------------------------------------------------------------ 806
PA14       ATCTTCACCGTGCTGACCTCCC-CCACCAGCGTCCATGGCCTGGCCAACATCGACTTCGT 932
R          ATCTTCACCGTGCTGACCTCCCCCCACCAGCGTCCATGGCCTGGCCAACATCGACTTCGT 494

F          ------------------------------------------------------------ 806
PA14       GATCTTCCCGCCGCGCTGGATGGTGGCCGAGAACACCTTCCGTCCGCCATGGTTCCACCG 992
R          GATCTTCCCGCCGCGCTGGATGGTGGCCGAGAACACCTTCCGTCCGCCATGGCTCCACCG 554

F          ------------------------------------------------------------ 806
PA14       CAACCTGATGAACGAGTTCATGGGCCTGATCCAGGGCGCCTATGACGCCAAGGCCGGCGG 1052
R          CAACCTGATGAACGAGTTCATGGGCCTGATCCAGGGCGCCTATGACGCCAAGGCCGGCGG 614

F          ------------------------------------------------------------ 806
PA14       CTTCGTGCCTGGCGGCGCCTCGCTGCACAGTTGCATGAGCGCCCACGGCCCGGACGCGGA 1112
R          CTTCGTGCCTGGCGGCGCCTCGCTGCACAGTTGCATGAGCGCCCACGGCCCGGACGCGGA 674

F          ------------------------------------------------------------ 806
PA14       AAGCTGCGACAAGGCCATCGCCGCCGACCTCAAGCCGCACAGGATCGACCAGACCATGGC 1172
R          AAGCTGCGACAAGGCCATCGCCGCCGACCTCAAGCCGCACAGGATCGACCAGACCATGGC 734

F          ------------------------------------------------------------ 806
PA14       CTTCATGTTCGAGACCAGCCAGGTCCTCCGGCCGAGCCGTGCCGCCCTCGAGACGCCGGC 1232
R          CTTCATGTTCGAGACCAGCCAGGTCCTCCGGCCGAGCCGTGCCGCCCTCGAGACGCCGGC 794

F          ------------------------------------------------------------ 806
PA14       CCTGCAGAATGACTACGATGCCTGCTGGGCGTCGCTCGTATCCACCTTCAACCCGCAACG 1292
R          CCTGCAGAATGACTACGATGCCTGCTGGGCGTCGCTCGTATCCACCTTCAACCCGCAACG 854

F          --------------------------------    806
PA14       GAGATAA-------------------------    1299
R          GAGATAACCCCATGAACCAGCCAACCCCCACT    886
```

Brown isolate 2

```
R       ------------------------------------------------------------ 0
PA14    -------------------------ATGAACCTCGACTCCACTGCCCTCGCCTATCAATC 35
F       CTGCCCCACTCCCCGGAGGCCTCAGATGAACCTCGACTCCACTGCCCTCGCCTATCAATC 60


R       ------------------------------------------------------------ 0
PA14    GGGCTTCGGCAACGAATTCAGCAGCGAAGCGCTCCCCGGCGCCCTGCCGGTCGGCCAGAA 95
F       GGGCTTCGGCAACGAATTCAGCAGCGAAGCGCTCCCCGGCGCCCTGCCGGTCGGCCAGAA 120


R       ------------------------------------------------------------ 0
PA14    CTCCCCGCAGAAAGCGCCCTACGGCCTGTACGCCGAACTGCTCTCCGGCACCGCCTTCAC 155
F       CTCCCCGCAGAAAGCGCCCTACGGCCTGTACGCCGAACTGCTCTCCGGCACCGCCTTCAC 180


R       ------------------------------------------------------------ 0
PA14    CATGGCTCGCAGCGAGGCCCGGCGCACCTGGCTATACCGCATCACGCCGTCGGCCAAGCA 215
F       CATGGCTCGCAGCGAGGCCCGGCGCACCTGGCTATACCGCATCACGCCGTCGGCCAAGCA 240


R       ------------------------------------------------------------ 0
PA14    TCCGCCGTTCCGCCGCCTGGAACGACAGATCGCCGGTGCCGAACTGGATGCGCCGACCCC 275
F       TCCGCCGTTCCGCCGCCTGGAACGACAGATCGCCGGTGCCGAACTGGATGCGCCGACCCC 300


R       ------------------------------------------------------------ 0
PA14    CAACCGCCTGCGCTGGGACCCGCTGGCACTGCCCGAGCAGCCCACCGACTTCCTCGACGG 335
F       CAACCGCCTGCGCTGGGACCCGCTGGCACTGCCCGAGCAGCCCACCGACTTCCTCGACGG 360


R       ------------------------------------------------------------ 0
PA14    CCTGCTGCGCATGGCCGCCAACGCGCCCGGCGACAAGCCCGCCGGCGTGAGCATCTACCA 395
F       CCTGCTGCGCATGGCCGCCAACGCGCCCGGCGACAAGCCCGCCGGCGTGAGCATCTACCA 420


R       ------------------------------------------------CGAACTGCTGCT 12
PA14    GTACCTGGCCAACCGCTCGATGGAGCGTTGCTTCTACGACGCCGACGGCGAACTGCTGCT 455
F       GTACCTGGCCAACCGCTCGATGGAGCGTTGCTTCTACGACGCCGACGGCGAACTGCTGCT 480
                                                        ************

R       GGTCCCGCAGTTGGGCCGCCTGCGCCTGTGCACCGAACTCGGCGCGCTGCAGGTCGAACC 72
PA14    GGTCCCGCAGTTGGGCCGCCTGCGCCTGTGCACCGAACTCGGCGCGCTGCAGGTCGAACC 515
F       GGTCCCGCAGTTGGGCCGCCTGCGCCTGTGCACCGAACTCGGCGCGCTGCAGGTCGAACC 540
        ************************************************************

R       GCTGGAGATCGCGGTGATCCCGCGCGGGATGAAGTTCCGCGTCGAGCTGCTCGACGGCGA 132
PA14    GCTGGAGATCGCGGTGATCCCGCGCGGGATGAAGTTCCGCGTCGAGCTGCTCGACGGCGA 575
F       GCTGGAGATCGCGGTGATCCCGCGCGGGATGAAGTTCCGCGTCGAGCTGCTCGACGGCGA 600
        ************************************************************

R       GGCACGCGGCTATATCGCCGAGAACCACGGCGCGCCGCTGCGCCTGCCCGACCTCGGCCC 192
PA14    GGCACGCGGCTATATCGCCGAGAACCACGGCGCGCCGCTGCGCCTGCCCGACCTCGGCCC 635
F       GGCACGCGGCTATATCGCCGAGAACCACGGCGCGCCGCTGCGCCTGCCCGACCTCGGCCC 660
        ************************************************************
```

```
R       GATCGGCAGCAATGGCCTGGCCAATCCGCGCGACTTCCTGGCCCCGGTGGCGCGCTACGA 252
PA14    GATCGGCAGCAATGGCCTGGCCAATCCGCGCGACTTCCTGGCCCCGGTGGCGCGCTACGA 695
F       GATCGGCAGCAATGGCCTGGCCAATCCGCGCGACTTCCTGGCCCCGGTGGCGCGCTACGA 720
        ************************************************************

R       AGACAGCCGCCAGCCGCTGCAACTGGTGCAGAAATACCTCGGCGAGCTGTGGGCCTGCGA 312
PA14    AGACAGCCGCCAGCCGCTGCAACTGGTGCAGAAATACCTCGGCGAGCTGTGGGCCTGCGA 755
F       AGACAGCCGCCAGCCGCTGCAACTGGTGCAGAAATACCTCGGCGAGCTGTGGGCCTGCGA 780
        ************************************************************

R       GCTTGACCACTCGCCGCTGGACGTGGTCGCCTGGCACGGCAACAACGTGCCCTACAAGTA 372
PA14    GCTTGACCACTCGCCGCTGGACGTGGTCGCCTGGCACGGCAACAACGTGCCCTACAAGTA 815
F       GCTTGACCACTCGCCGCTGGACGTGGTCGCCTGGCACGGCAACAACGTGCCCTACAAGTA 840
        ************************************************************

R       CGACCTGCGCCGCTTCAACACCATCGGCACGGTCAGCTTCGACCACCCGGACCCGTCGAT 432
PA14    CGACCTGCGCCGCTTCAACACCATCGGCACGGTCAGCTTCGACCACCCGGACCCGTCGAT 875
F       CGACCTGCGCCGCTTCAACACCATCGGCACGGTCAGCTTCGACCACCCGGACCCGTCGAT 900
        ************************************************************

R       CTTCACCGTGCTGACCTCCCCCACCAGCGTCCATGGCCTGGCCAACATCGACTTCGTGAT 492
PA14    CTTCACCGTGCTGACCTCCCCCACCAGCGTCCATGGCCTGGCCAACATCGACTTCGTGAT 935
F       CTTCACCGTGCTGACCTCCCCCACCAGCGTCCATGGCCTGGCCAACATCGACTTCGTGAT 960
        ************************************************************

R       CTTCCCGCCGCGCTGGATGGTGGCCGAGAACACCTTCCGTCCGCCATGGCTCCACCGCAA 552
PA14    CTTCCCGCCGCGCTGGATGGTGGCCGAGAACACCTTCCGTCCGCCATGGTTCCACCGCAA 995
F       CTC--------------------------------------------------------- 963
        **

R       CCTGATGAACGAGTTCATGGGCCTGATCCAGGGCGCCTATGACGCCAAGGCCGGCGGCTT 612
PA14    CCTGATGAACGAGTTCATGGGCCTGATCCAGGGCGCCTATGACGCCAAGGCCGGCGGCTT 1055
F       ------------------------------------------------------------ 963

R       CGTGCCTGGCGGCGCCTCGCTGCACAGTTGCATGAGCGCCCACGGCCCGGACGCGGAAAG 672
PA14    CGTGCCTGGCGGCGCCTCGCTGCACAGTTGCATGAGCGCCCACGGCCCGGACGCGGAAAG 1115
F       ------------------------------------------------------------ 963

R       CTGCGACAAGGCCATCGCCGCCGACCTCAAGCCGCACAGGATCGACCAGACCATGGCCTT 732
PA14    CTGCGACAAGGCCATCGCCGCCGACCTCAAGCCGCACAGGATCGACCAGACCATGGCCTT 1175
F       ------------------------------------------------------------ 963

R       CATGTTCGAGACCAGCCAGGTCCTCCGGCCGAGCCGTGCCGCCCTCGAGACGCCGGCCCT 792
PA14    CATGTTCGAGACCAGCCAGGTCCTCCGGCCGAGCCGTGCCGCCCTCGAGACGCCGGCCCT 1235
F       ------------------------------------------------------------ 963

R       GCAGAATGACTACGATGCCTGCTGGGCGTCGCTCGTATCCACCTTCAACCCGCAACGGAG 852
PA14    GCAGAATGACTACGATGCCTGCTGGGCGTCGCTCGTATCCACCTTCAACCCGCAACGGAG 1295
F       ------------------------------------------------------------ 963
```

```
R          ATAACCCCATGAACCAGCCAACCCCCACTGAG    884
PA14       ATAA----------------------------    1299
F          --------------------------------    963
```

Brown isolate 3

```
F          TGCCCCACTCCCCGGAGGCCTCAGATGAACCTCGACTCCACTGCCCTCGCCTATCAATCG 60
PA14       ------------------------ATGAACCTCGACTCCACTGCCCTCGCCTATCAATCG 36
R          ------------------------------------------------------------ 0


F          GGCTTCGGCAACGAATTCAGCAGCGAAGCGCTCCCCGGCGCCCTGCCGGTCGGCCAGAAC 120
PA14       GGCTTCGGCAACGAATTCAGCAGCGAAGCGCTCCCCGGCGCCCTGCCGGTCGGCCAGAAC 96
R          ------------------------------------------------------------ 0


F          TCCCCGCAGAAAGCGCCCTACGGCCTGTACGCCGAACTGCTCTCCGGCACCGCCTTCACC 180
PA14       TCCCCGCAGAAAGCGCCCTACGGCCTGTACGCCGAACTGCTCTCCGGCACCGCCTTCACC 156
R          ------------------------------------------------------------ 0


F          ATGGCTCGCAGCGAGGCCCGGCGCACCTGGCTATACCGCATCACGCCGTCGGCCAAGCAT 240
PA14       ATGGCTCGCAGCGAGGCCCGGCGCACCTGGCTATACCGCATCACGCCGTCGGCCAAGCAT 216
R          ------------------------------------------------------------ 0


F          CCGCCGTTCCGCCGCCTGGAACGACAGATCGCCGGTGCCGAACTGGATGCGCCGACCCCC 300
PA14       CCGCCGTTCCGCCGCCTGGAACGACAGATCGCCGGTGCCGAACTGGATGCGCCGACCCCC 276
R          ------------------------------------------------------------ 0


F          AACCGCCTGCGCTGGGACCCGCTGGCACTGCCCGAGCAGCCCACCGACTTCCTCGACGGC 360
PA14       AACCGCCTGCGCTGGGACCCGCTGGCACTGCCCGAGCAGCCCACCGACTTCCTCGACGGC 336
R          ------------------------------------------------------------ 0


F          CTGCTGCGCATGGCCGCCAACGCGCCCGGCGACAAGCCCGCCGGCGTGAGCATCTACCAG 420
PA14       CTGCTGCGCATGGCCGCCAACGCGCCCGGCGACAAGCCCGCCGGCGTGAGCATCTACCAG 396
R          -------------------------------------------------GCATTTACCAG 11
                                                            **** ******

F          TACCTGGCCAACCGCTCGATGGAGCGTTGCTTCTACGACGCCGACGGCGAACTGCTGCTG 480
PA14       TACCTGGCCAACCGCTCGATGGAGCGTTGCTTCTACGACGCCGACGGCGAACTGCTGCTG 456
R          TACCTGGCCAACCGCTCGATGGAGCGT-TGTTCTACGACGCCGACGGCGAACTGCTGCTG 70
           ***************************   ******************************

F          GTCCCGCAGTTGGGCCGCCTGCGCCTGTGCACCGAACTCGGCGCGCTGCAGGTCGAACCG 540
PA14       GTCCCGCAGTTGGGCCGCCTGCGCCTGTGCACCGAACTCGGCGCGCTGCAGGTCGAACCG 516
R          GTCCCGCAGTTGGGCCGCCTGCGCCTGTGCACCGAACTCGGCGCGCTGCAGGTCGAACCG 130
           ************************************************************

F          CTGGAGATCGCGGTGATCCCGCGCGGGATGAAGTTCCGCGTCGAGCTGCTCGACGGCGAG 600
PA14       CTGGAGATCGCGGTGATCCCGCGCGGGATGAAGTTCCGCGTCGAGCTGCTCGACGGCGAG 576
R          CTGGAGATCGCGGTGATCCCGCGCGGGATGAAGTTCCGCGTCGAGCTGCTCGACGGCGAG 190
           ************************************************************

F          GCACGCGGCTATATCGCCGAGAACCACGGCGCGCCGCTGCGCCTGCCCGACCTCGGCCCG 660
```

```
PA14    GCACGCGGCTATATCGCCGAGAACCACGGCGCGCCGCTGCGCCTGCCCGACCTCGGCCCG 636
R       GCACGCGGCTATATCGCCGAGAACCACGGCGCGCCGCTGCGCCTGCCCGACCTCGGCCCG 250
        ************************************************************

F       ATCGGCAGCAATGGCCTGGCCAATCCGCGCGACTTCCTGGCCCCCGGTGGCGCGCTACGA 720
PA14    ATCGGCAGCAATGGCCTGGCCAATCCGCGCGACTTCCTGGCC-CCGGTGGCGCGCTACGA 695
R       ATCGGCAGCAATGGCCTGGCCAATCCGCGCGACTTCCTGGCC-CCGGTGGCGCGCTACGA 309
        ****************************************** *****************

F       AGACAGCCGCCAGCCGCTGCAACTGGTGCAGAAATACCTCGGCGGAGCTGTGGGCCTGCG 780
PA14    AGACAGCCGCCAGCCGCTGCAACTGGTGCAGAAATACCTCGGCGA-GCTGTGGGCCTGCG 754
R       AGACAGCCGCCAGCCGCTGCAACTGGTGCAGAAATACCTCGGCGA-GCTGTGGGCCTGCG 368
        ********************************************  **************

F       AGCTTGACCACTCGCCGCTTGGACGTGGTCGCCTGGCACGGCAACAACGTGCCCTACAAG 840
PA14    AGCTTGACCACTCGCC-GCTGGACGTGGTCGCCTGGCACGGCAACAACGTGCCCTACAAG 813
R       AGCTTGACCACTCGCC-GCTGGACGTGGTCGCCTGGCACGGCAACAACGTGCCCTACAAG 427
        ****************   *****************************************

F       TACGACCTGCGCCGCTTCAACACCATCGGCACGGTCAGCTTCGACCACCCGGACCCGTCG 900
PA14    TACGACCTGCGCCGCTTCAACACCATCGGCACGGTCAGCTTCGACCACCCGGACCCGTCG 873
R       TACGACCTGCGCCGCTTCAACACCATCGGCACGGTCAGCTTCGACCACCCGGACCCGTCG 487
        ************************************************************

F       ATCTTCACCGTGCTGACC------------------------------------------ 918
PA14    ATCTTCACCGTGCTGACCTCCCCCACCAGCGTCCATGGCCTGGCCAACATCGACTTCGTG 933
R       ATCTTCACCGTGCTGACCTCCCCCACCAGCGTCCATGGCCTGGCCAACATCGACTTCGTG 547
        ******************

F       ------------------------------------------------------------ 918
PA14    ATCTTCCCGCCGCGCTGGATGGTGGCCGAGAACACCTTCCGTCCGCCATGGTTCCACCGC 993
R       ATCTTCCCGCCGCGCTGGATGGTGGCCGAGAACACCTTCCGTCCGCCATGGCTCCACCGC 607

F       ------------------------------------------------------------ 918
PA14    AACCTGATGAACGAGTTCATGGGCCTGATCCAGGGCGCCTATGACGCCAAGGCCGGCGGC 1053
R       AACCTGATGAACGAGTTCATGGGCCTGATCCAGGGCGCCTATGACGCCAAGGCCGGCGGC 667

F       ------------------------------------------------------------ 918
PA14    TTCGTGCCTGGCGGCGCCTCGCTGCACAGTTGCATGAGCGCCCACGGCCCGGACGCGGAA 1113
R       TTCGTGCCTGGCGGCGCCTCGCTGCACAGTTGCATGAGCGCCCACGGCCCGGACGCGGAA 727

F       ------------------------------------------------------------ 918
PA14    AGCTGCGACAAGGCCATCGCCGCCGACCTCAAGCCGCACAGGATCGACCAGACCATGGCC 1173
R       AGCTGCGACAAGGCCATCGCCGCCGACCTCAAGCCGCACAGGATCGACCAGACCATGGCC 787

F       ------------------------------------------------------------ 918
PA14    TTCATGTTCGAGACCAGCCAGGTCCTCCGGCCGAGCCGTGCCGCCCTCGAGACGCCGGCC 1233
R       TTCATGTTCGAGACCAGCCAGGTCCTCCGGCCGAGCCGTGCCGCCCTCGAGACGCCGGCC 847

F       ------------------------------------------------------------ 918
PA14    CTGCAGAATGACTACGATGCCTGCTGGGCGTCGCTCGTATCCACCTTCAACCCGCAACGG 1293
```

```
R         CTGCAGAATGACTACGATGCCTGCTGGGCGTCGCTCGTATCCACCTTCAACCCGCAACGG 907


F         ----------------------------------- 918
PA14      AGATAA----------------------------- 1299
R         AGATAACCCCATGAACCAGCCAACCCCCACTCGTG 942
```
